# Exacerbated Innate Host Response to SARS-CoV in Aged Non-Human Primates

**DOI:** 10.1371/journal.ppat.1000756

**Published:** 2010-02-05

**Authors:** Saskia L. Smits, Anna de Lang, Judith M. A. van den Brand, Lonneke M. Leijten, Wilfred F. van IJcken, Marinus J. C. Eijkemans, Geert van Amerongen, Thijs Kuiken, Arno C. Andeweg, Albert D. M. E. Osterhaus, Bart L. Haagmans

**Affiliations:** 1 Department of Virology, Erasmus Medical Center, Rotterdam, The Netherlands; 2 Erasmus Center for Biomics, Erasmus Medical Center, Rotterdam, The Netherlands; 3 Department of Public Health, Erasmus Medical Center, Rotterdam, The Netherlands; University of North Carolina, United States of America

## Abstract

The emergence of viral respiratory pathogens with pandemic potential, such as severe acute respiratory syndrome coronavirus (SARS-CoV) and influenza A H5N1, urges the need for deciphering their pathogenesis to develop new intervention strategies. SARS-CoV infection causes acute lung injury (ALI) that may develop into life-threatening acute respiratory distress syndrome (ARDS) with advanced age correlating positively with adverse disease outcome. The molecular pathways, however, that cause virus-induced ALI/ARDS in aged individuals are ill-defined. Here, we show that SARS-CoV-infected aged macaques develop more severe pathology than young adult animals, even though viral replication levels are similar. Comprehensive genomic analyses indicate that aged macaques have a stronger host response to virus infection than young adult macaques, with an increase in differential expression of genes associated with inflammation, with NF-κB as central player, whereas expression of type I interferon (IFN)-β is reduced. Therapeutic treatment of SARS-CoV-infected aged macaques with type I IFN reduces pathology and diminishes pro-inflammatory gene expression, including interleukin-8 (IL-8) levels, without affecting virus replication in the lungs. Thus, ALI in SARS-CoV-infected aged macaques developed as a result of an exacerbated innate host response. The anti-inflammatory action of type I IFN reveals a potential intervention strategy for virus-induced ALI.

## Author Summary

Severe acute respiratory syndrome coronavirus (SARS-CoV) infection causes acute lung injury that may develop into the life-threatening acute respiratory distress syndrome (ARDS) in mostly elderly individuals. Although SARS-CoV infection can be fatal, most patients recover, suggesting that protective host responses are operational to combat the viral infection. Therefore, we used age as predisposing factor to obtain insight into the pathogenesis of SARS-CoV. In this study, we show that SARS-CoV-infected aged macaques developed significantly more pathology than young adult animals, which could not be contributed to differences in viral replication. Using comparative microarray analyses, it was shown that although the nature of the host response to SARS-CoV infection was similar in aged and young adult macaques, the severity was significantly different, with aged macaques displaying an increase in differential expression of genes associated with inflammation. Interestingly, type I IFN-β mRNA levels correlated negatively with gross pathology. Therapeutic treatment of aged macaques with type I IFN reduced pathology without affecting virus replication. However, pro-inflammatory gene expression was significantly diminished. Thus, modulation of the host response by type I IFNs provides a promising outlook for novel intervention strategies.

## Introduction

The zoonotic transmission of severe acute respiratory syndrome coronavirus (SARS-CoV) caused pneumonic disease in humans with an overall mortality rate of ∼10%. The exact reasons why some individuals succumbed to the infection while others remained relatively unaffected have not been clarified. Aging, an important risk factor in SARS-CoV-associated disease, is associated with changes in immunity [1],[2],[3]. Consequently, elderly individuals are at greater risk of contracting more severe and longer lasting infections with increased morbidity and mortality, exemplified by respiratory tract infections caused by influenza A virus and severe acute respiratory syndrome (SARS) coronavirus [4],[5],[6]. The clinical course of SARS-CoV-induced disease follows a triphasic pattern [5]. The first phase is characterized by fever, myalgia and other systemic symptoms that are likely caused by the increase in viral replication and cytolysis. The second phase of the disease is characterized by a decrease in viral replication that correlates with the onset of IgG conversion. Interestingly, it is also in this phase that severe clinical worsening is seen, which can not be explained by uncontrolled viral replication. It has been hypothesized that the diffuse alveolar lung damage in this phase is caused by an over exuberant host response [5],[7],[8]. The majority of patients recovers after 1–2 weeks, but up to one-third of the patients progress to the third phase and develop severe inflammation of the lung, characterized by acute respiratory distress syndrome (ARDS) [9]. The clinical course and outcome of SARS-CoV disease are more favorable in children younger than 12 years of age as compared to adolescents and adults [10],[11],[12]; elderly patients have a poor prognosis, with mortality rates of up to ∼50% [5],[6].

For SARS-CoV-associated disease in humans, it has been hypothesized that seemingly excessive pro-inflammatory responses, illustrated by elevated levels of inflammatory cytokines and chemokines, mediate immune-pathology resulting in acute lung injury (ALI) and ARDS [5],[13],[14],[15],[16]. Direct support for this concept, however, is scarce. ALI and ARDS are typified by inflammation, with increased permeability of the alveolar-capillary barrier, resulting in pulmonary edema, hypoxia, and accumulation of polymorphonuclear leukocytes and macrophages. Inflammatory cytokines, among which IL-1β and IL-8, play a major role in mediating and amplifying ALI/ARDS [9] and are elevated in SARS-CoV-infected patients as well [13],[14]. *In vitro* experiments confirm that SARS-CoV infection induces expression of cytokines/chemokines in a range of cell types [15],[17],[18]. Moreover, infection of cynomolgus macaques with SARS-CoV leads to a strong immune response, with expression of various cytokines/chemokines, resembling the host response seen in human SARS patients [19]. Nevertheless, the determinants that lead to severe virus-associated ALI/ARDS and that cause people to succumb to infection remain largely obscure, restraining development of appropriate treatments.

As advanced age is a predictor of adverse clinical outcome in both ARDS and SARS-CoV infections [5],[20], we used age as predisposing factor to study the pathogenesis of SARS-CoV in a macaque model. By performing comparative analyses of young adult and aged SARS-CoV-infected macaques regarding pathology, virus replication and host response, insight into the pathogenesis of SARS-CoV is obtained and a potential therapeutic intervention strategy for virus-induced ALI is revealed.

## Results

### SARS-CoV causes more severe pathology in aged than in young adult macaques

**Figure 1:**
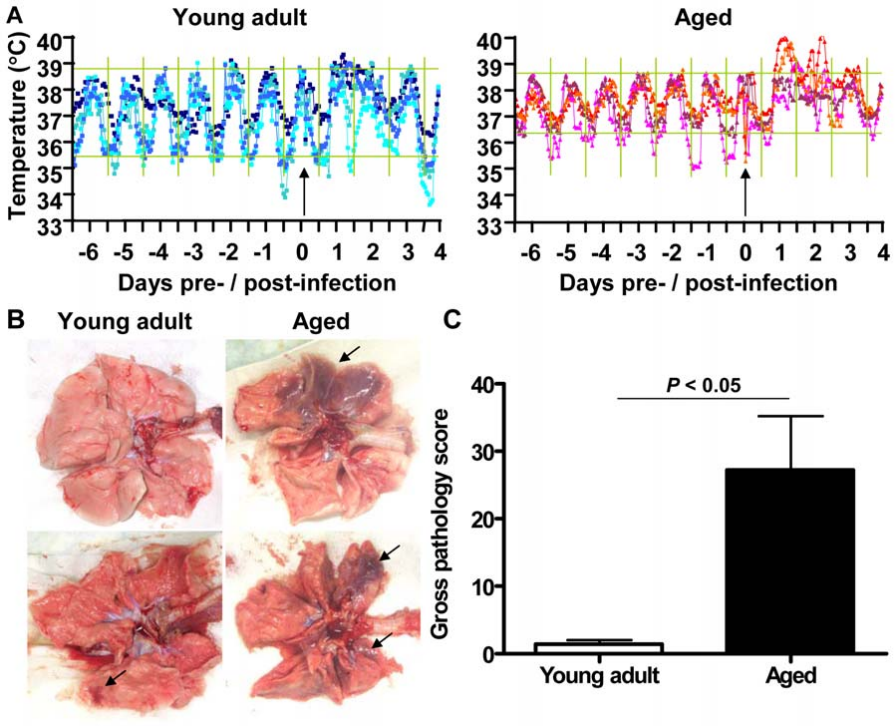
Aged macaques are more prone to develop SARS-CoV-associated disease than young adults. (A) Fluctuations in body temperatures in four young adult and four aged SARS-CoV-infected macaques measured by transponders in the peritoneal cavity. Temperatures are shown from day six prior to infection until four days post infection. The arrow indicates day zero when animals were infected. Grey horizontal lines mark the average range of temperature fluctuations prior to infection. (B) Macroscopic appearance of (consolidated) lung tissue of young adult and aged SARS-CoV infected macaques at day 4 post infection. Lesions are arrowed. (C) Gross pathology scores of aged and young adult macaque groups were determined after necropsy and averaged (±standard error of the mean (s.e.m.)).

**Figure 2:**
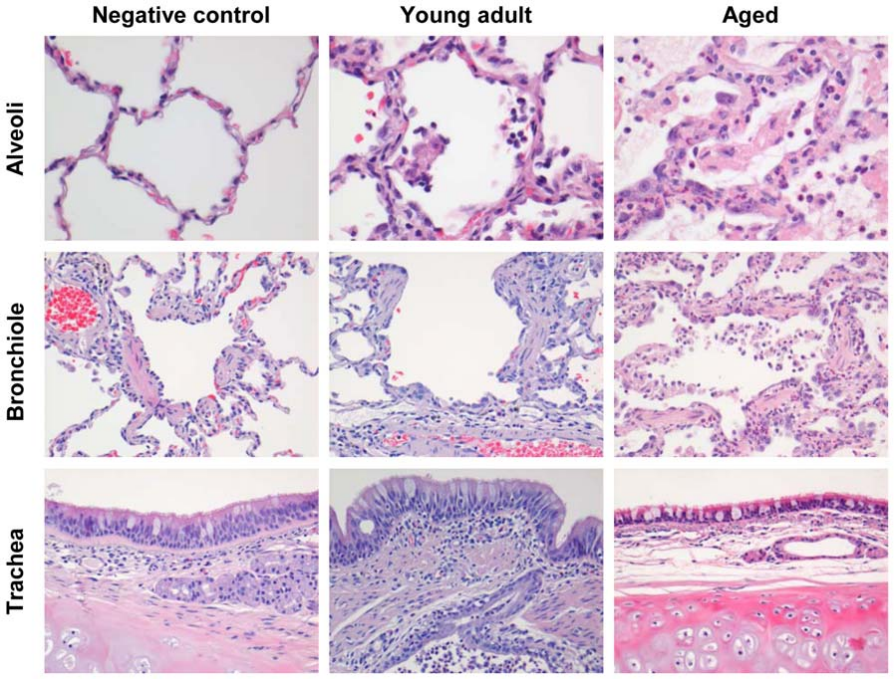
Histology of lungs from SARS-CoV-infected aged macaques. Lesions in lungs of PBS-infected (left panel) and SARS-CoV infected young adult (middle panel) and aged (right panel) macaques showing diffuse alveolar damage, characterized by disruption of alveolar walls causing edema and type II pneumocyte hyperplasia with influx of inflammatory cells in the alveoli and bronchioles. In the trachea, a multifocal mild chronic lymphoplasmacytic tracheobronchoadenitis was observed in young adult macaques.

To obtain further insights in the pathogenesis of SARS-CoV, six aged (10–19 years old) and six young adult (3–5 years old) cynomolgus macaques were infected with SARS-CoV HKU39849 and euthanized four days after infection. Four young adult and four aged PBS-infected cynomolgus macaques were used as negative controls. During the 4-day experiment, some of the SARS-CoV-infected aged animals displayed decreased activity and mildly labored breathing. All aged infected macaques showed an increase in body temperature either during the night or during the day one to two days after infection (Figure 1A). The lungs of aged macaques showed large (multi)focal pulmonary consolidation that was severe (∼40–60% of affected lung tissue) in two macaques (Figure 1B and Figure S1). Microscopic examination revealed typical ALI-associated lesions, similar to what has been seen in SARS-CoV-infected humans that progress to ARDS [6]. Lesions involved the alveoli and terminal bronchioli, showing areas with acute or more advanced phases of diffuse alveolar damage (Figure 2). Lumina of alveoli were variably filled with protein rich edema fluid, cellular debris, alveolar macrophages and neutrophils, eosinophils, and lymphocytes (Figure 2 and Figure S2A–B). Moderately thickened alveolar walls were lined by cuboidal epithelial cells (type 2 pneumocyte hyperplasia; Figure 2 and Figure S2C). The epithelial origin of these enlarged type 2 pneumocytes with large vacuolated nuclei, prominent nucleoli and abundant vesicular cytoplasm was confirmed by keratin staining (Figure S2D). Hyaline membranes and multinucleated giant cells were occasionally observed in the alveoli (Figure S2E–F). In contrast, all young adult animals remained free of clinical symptoms and had no or less extensive pulmonary consolidation (Figure 1A–C). Hyaline membranes were not observed in SARS-CoV-infected young adult macaques. A multifocal mild chronic lymphoplasmacytic tracheo-bronchoadenitis, characterized by moderate numbers of lymphocytes, plasma cells, macrophages, less neutrophils and occasional eosinophils in the lamina propria of the bronchi, focally surrounding and infiltrating the submucosal glands, was observed in all young adult macaques, but not in aged macaques (Figure 2). Our data were confirmed by retrospective analysis of earlier experiments in which aged animals were used [19],[21],[22],[23]. Overall, aged macaques develop more severe SARS-CoV-associated ALI than young adults.

### The level of viral replication in aged and young adult macaques is similar

**Figure 3:**
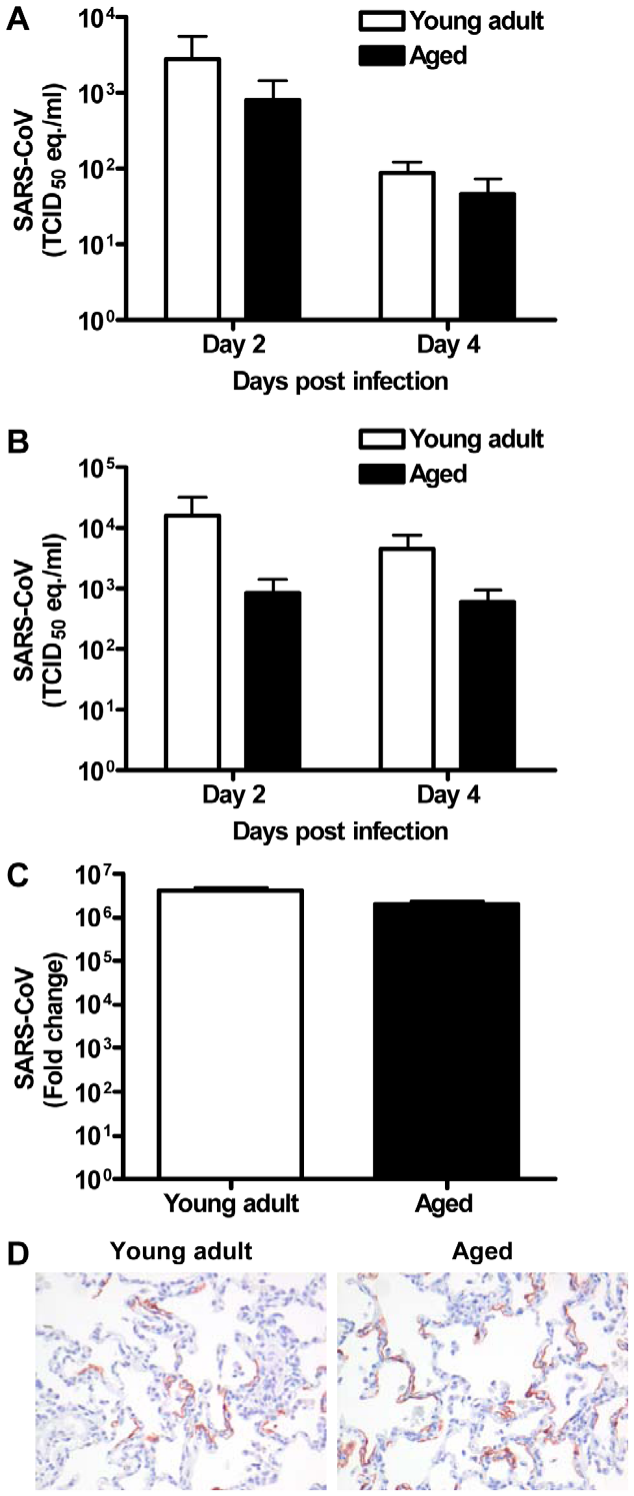
Viral replication levels in SARS-CoV-infected aged and young adult macaques are similar. (A–B) SARS-CoV replication in the throat (A) and nose (B) of SARS-CoV infected aged (black bars) and young adult (white bars) macaques at day 2 and 4 post infection as determined by real-time RT-PCR. Viral RNA levels are displayed as TCID_50_ equivalents (eq.)/ml swab medium (±s.e.m.). (C) Average fold change in SARS-CoV mRNA levels (±s.e.m.) in the lungs of aged and young adult macaques compared to PBS-infected animals as determined by real-time RT-PCR and depicted on a log-scale. (D) Lung sections of SARS-CoV-infected aged and young adult macaques were stained with a mouse-anti-SARS-nucleocapsid IgG2a. Sections were counterstained with hematoxylin. Original magnifications are ×20.

Because viral replication is important for disease pathogenesis, we determined virus titers in aged and young adult animals. Virus excretion in the throat (Figure 3A) and nose (Figure 3B) of aged and young adult macaques at days 2 and 4 post infection was not significantly different. Moreover, no significant difference in quantity of SARS-CoV mRNA in the lungs of young adult and aged animals was observed (Figure 3C). Differences in the nature and percentage of SARS-CoV-infected cells in the lungs of aged and young adult macaques were not seen either (Figure 3D). Apparently, augmented pathology in aged macaques cannot be rationalized by increased viral replication.

### The host response to SARS-CoV infection is stronger in aged than in young adult macaques

**Figure 4:**
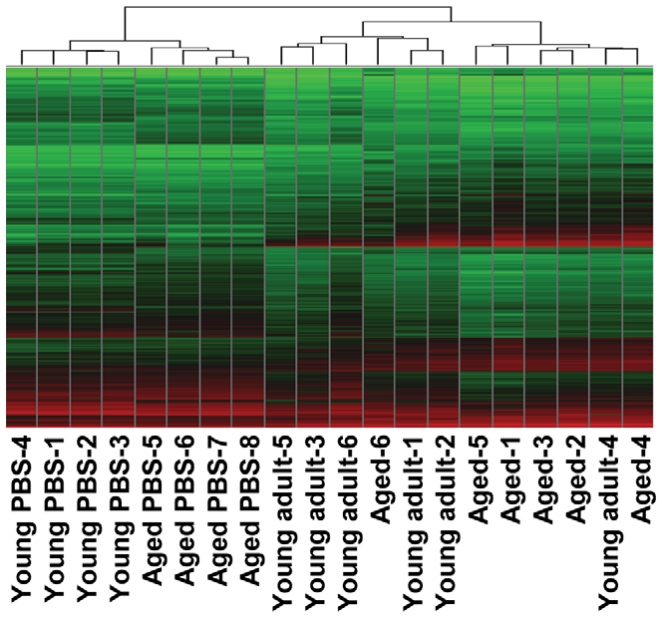
Global gene expression profiles of individual young adult and aged animals. Global gene expression profiles of normalized log-2 based hybridization signals of individual young adult and aged macaques of a set of gene transcripts that were identified as being differentially regulated (fold change ≥2; FDR<0.05) in at least one of the comparisons of gene expression in the lungs of experimentally SARS-CoV-infected aged and young adult macaques versus gene expression in the lungs of PBS-infected macaques, and genes were included if they met the criteria of an absolute fold change of ≥2-fold (FDR<0.05) in at least one experiment. The data were plotted as a heat map, where each matrix entry represents a gene expression value. Normalized log-2 based hybridization signals ranged from 3 (green) to 14 (red). Dendograms (trees) of the heat map represent the degree of relatedness between the samples, with short branches denoting a high degree of similarity and long branches denoting a low degree of similarity.

To understand why SARS-CoV-infection in aged macaques results in more severe pathology than in young adult macaques, we determined global gene expression profiles by analyzing total RNA isolated from the lungs using microarray analysis. Hierarchical clustering methods were used to order gene transcripts and individual aged and young adult animals to identify groups of animals with similar expression patterns. These data were plotted as a heat map in which each entry represents a gene expression value (Figure 4). As expected in an animal experiment with outbred animals, the inter-animal variation was relatively high (Figure S3). There were two major roots to the hierarchical dendogram, with one root containing the PBS-infected control animals, and the second root containing the SARS-CoV-infected animals. The root of the PBS-infected control animals was divided in two minor roots, clustering young adults together and aged animals as a group. These data suggest that the baseline expression patterns are different in young adult and aged macaques. The root of the SARS-CoV-infected animals was also divided in two minor roots, largely clustering young adult animals together and grouping aged infected macaques. The hierarchical clustering heat map suggests that both age and SARS-CoV infection are key factors involved in determining transcription of cellular genes.

**Figure 5:**
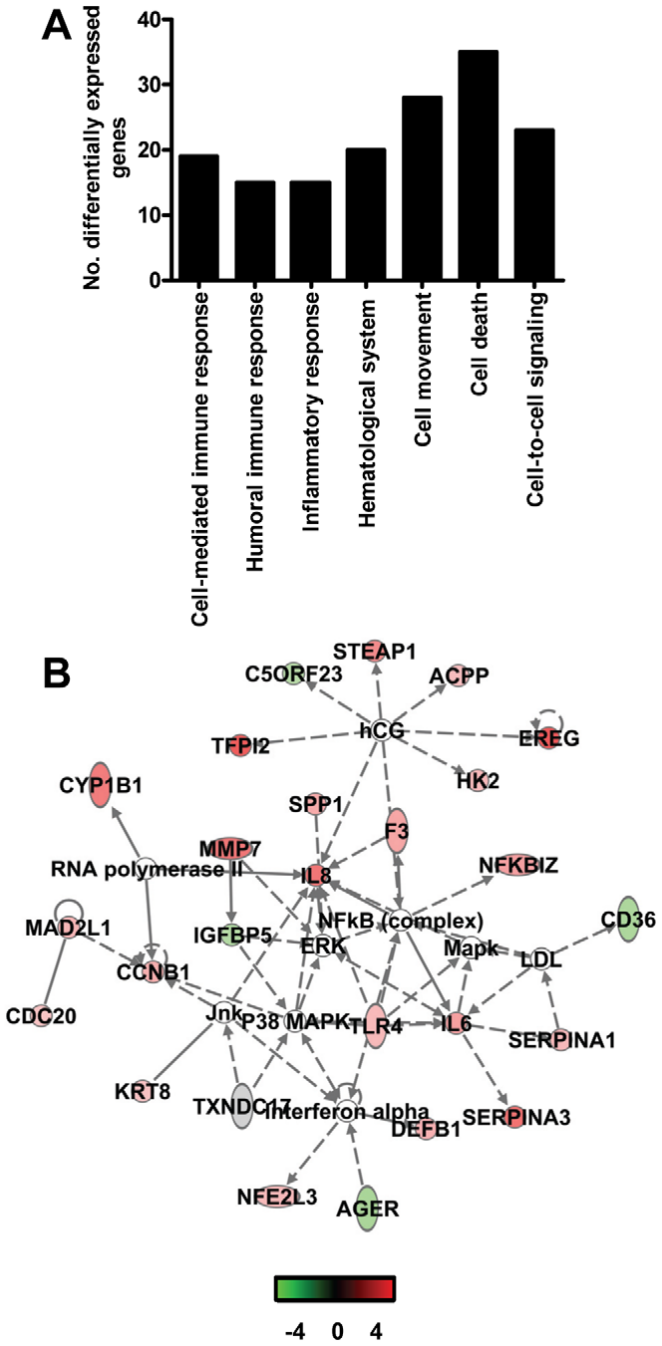
Direct comparison of gene expression profiles in the lungs of aged and young adult SARS-CoV-infected macaques. (A) Number of differentially expressed genes in the direct contrast of aged and young adult SARS-CoV-infected macaques with functions in immune response, inflammatory response, hematological system development and function, cell movement, cell death, or cell-to-cell signaling and interaction obtained from Ingenuity Pathways Knowledge Base. (B) This diagram shows a gene interaction network from Ingenuity Pathways Knowledge Base with genes that are differentially expressed in the contrast of aged and young adult SARS-CoV-infected animals. The central node is NF-κB, a key transcription factor in inflammation and ARDS. Genes depicted in green are downregulated and in red upregulated.

To determine whether aged and young adult animals respond differently to SARS-CoV-infection, their gene expression profiles were compared. In a direct comparison of aged (*n* = 6) versus young adult (*n* = 6) SARS-CoV-infected animals using an ANOVA-based analysis called LIMMA, 202 gene transcripts were differentially expressed (fold change ≥2; *p*<0.05; Table S1). Upon analysis of these gene transcripts within the context of biological processes and pathways using Ingenuity Pathways Knowledge Base, this subset of genes showed indications for an innate host response to viral infection. Among the top significantly differentially regulated (*p*<0.005) functional categories were immune response, inflammatory response and hematological system development and function, which included genes like *F3*, *IL1RL1*, *IL1RN*, *IL6*, *IL8*, *S100A8*, *SERPINA1*, *SERPINA3*, *NP*, *ACPP*, *TFPI2*, *SPP1*, *IGF1*, *EDN3*, *DEFB1*, and *SOCS3* (Figure 5A) most of which were upregulated in SARS-CoV-infected aged animals compared to young adult infected animals. In addition, three of the most significantly regulated molecular/cellular functions (*p*<0.005) were associated with a pro-inflammatory response and included cell death, cell movement, and cell-to-cell signalling (Figure 5A). The top gene interaction network, showing the interplay between genes during the host response to viral infection, contained NF-κB as central node (Figure 5B). NF-κB is a redox-sensitive transcription factor implicated to play a major role in pro-inflammatory host responses and the development of ALI/ARDS [24],[25]. Several of the 202 differentially expressed gene transcripts, among which *IL1RN*, *SERPINA1*, *IL8*, *F3* and *TFPI2*, are target genes for NF-κB. Thus, significant differences exist in the host response to SARS-CoV infection, corresponding with age.

**Figure 6:**
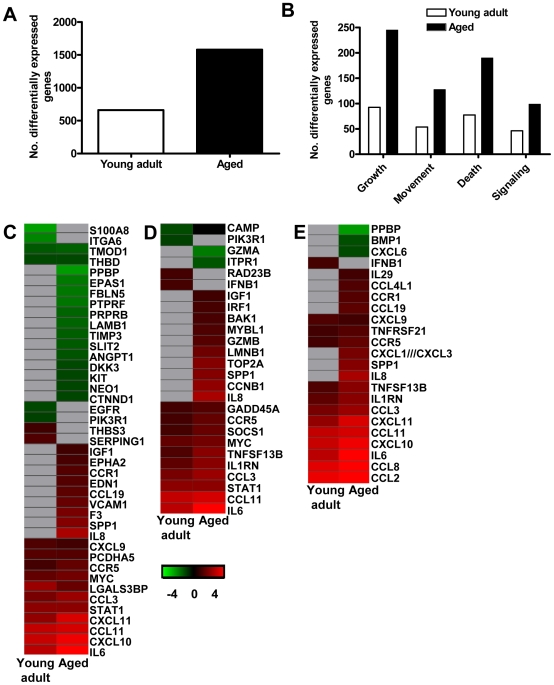
Aged macaques display a stronger host response to SARS-CoV infection than young adults. (A) Number of differentially expressed gene transcripts in aged and young adult SARS-CoV-infected macaques compared to aged and young adult PBS-infected animals, respectively (≥2-fold change, FDR<0.05, LIMMA analysis). (B) Number of differentially expressed genes in aged and young adult macaque groups compared to aged and young adult PBS-infected animals, respectively, with functions in cellular growth and proliferation, cell movement, cell death, or cell-to-cell signaling and interaction obtained from Ingenuity Pathways Knowledge Base. White bars for young adult and black bars for aged macaques. When SARS-CoV-infected aged and young adult macaques were compared directly, these cellular functions were significantly differentially expressed as well. (C–E) Gene expression profiles showing differentially expressed genes coding for proteins involved in cell adhesion (C), apopotosis (D), and cytokine/chemokine signalling (E) of aged and young adult macaques. Gene sets were obtained from Ingenuity Pathways Knowledge Base and changed ≥2-fold in at least one of the macaque groups as compared to PBS-infected controls. The data presented are error-weighted fold change averages for six young adult and aged animals. Genes shown in red were upregulated, in green downregulated, and in grey not significantly diferentially expressed in infected animals relative to PBS-infected animals (log (base 2) transformed expression values with minimum and maximum values of the color range being −4 and 4). Global test analysis of the direct contrast of SARS-CoV-infected aged versus young adult animals showed that these pathways were significantly differentially expressed (*P*<0.05). See Table S2 and S3 for full gene names and expression values.

To obtain a more in-depth view of the host response to infection, global gene expression profiles were determined in lungs of SARS-CoV-infected aged (*n* = 6) or young adult (*n* = 6) macaques in comparison to aged or young adult PBS-infected macaques (*n* = 4), respectively. Aged macaques differentially expressed 1577 gene transcripts (Figure 6A). Gene ontology analysis revealed that the majority of genes in the aged macaque group compared to aged PBS-infected animals were associated with a pro-inflammatory response and included cellular growth and proliferation, cell death, cell movement, and cell-to-cell signalling (Figure 6B). Although SARS-CoV-infected young adult macaques differentially expressed much less gene transcripts compared to young adult PBS-infected animals (Figure 6A), the most significantly regulated molecular/cellular functions also included cellular growth and proliferation, cell death, cell movement, and cell-to-cell signalling (Figure 6B). This suggested that the nature of the host response to infection in aged and young adult animals was strikingly similar, even though the severity was different.

Because the above described gene ontology molecular/cellular functions are very broad, genes were further subdivided based on available annotations to gain insight in differences in the host response to infection in aged and young adult macaques compared to aged and young adult PBS-infected animals, respectively. Heat maps were generated for differentially regulated genes with pro-inflammatory functions such as cell adhesion (Figure 6C), apoptosis (Figure 6D), and cytokine/chemokine signalling (Figure 6E). The greater number of differentially expressed genes, as well as the brighter intensities (fold changes in transcripts) included in the heat map for aged macaques, suggested that aged macaques show a more zealous response to virus infection than young adult macaques. This assumption was corroborated using Goeman's global test [26] on the defined gene subsets cell adhesion, cytokine/chemokine signalling, and apoptosis. When macaques were grouped according to severity of pathology instead of age and compared to their respective PBS-infected controls, increased numbers of differentially expressed gene transcripts and increased fold changes for differentially expressed genes in inflammatory pathways correlated positively with gross pathology scores as well (Figure S4). Our data show that the innate host response to SARS-CoV infection changes during aging in macaques; age, pathology, and pro-inflammatory host response go hand-in-hand.

In order to understand the host responses in the context of senescence, we directly compared lung samples from PBS-infected aged (*n* = 4) and young adult (*n* = 4) macaques. LIMMA analysis revealed that 518 gene transcripts were differentially expressed (fold change ≥2; *p*<0.05), with categories such as immunological disease, haematological system and development, cell death, cell movement, and cellular growth and proliferation among the top significantly differentially regulated functions (*p*<0.005). Only 14 out of the 518 differentially expressed gene transcripts were also differentially expressed in the direct contrast of SARS-CoV-infected aged and young adult macaques. Our data indicate that significant differences exist in the basal gene expression levels of aged and young adult macaques, which may partly explain why differences in pathology were observed after SARS-CoV infection.

### NF-κB signalling in SARS-CoV-infected macaques

**Figure 7:**
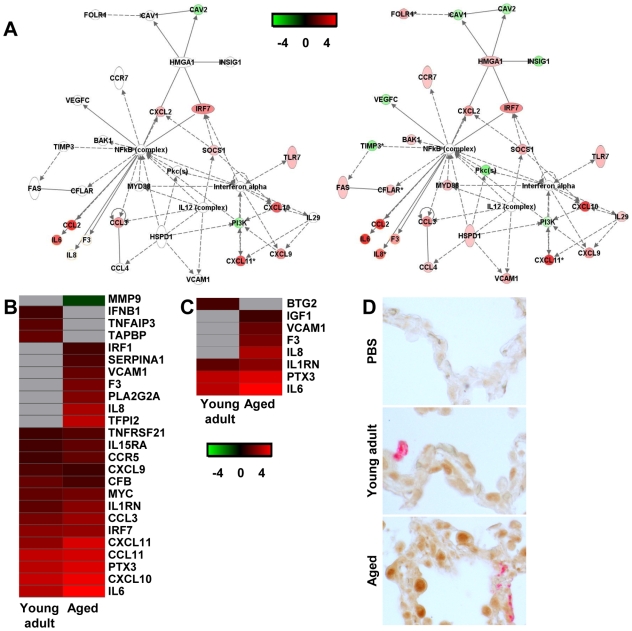
NF-κB-signalling in aged and young adult macaques. (A) These diagrams show a gene interaction network from Ingenuity Pathways Knowledge Base with genes that are differentially expressed in the contrast of aged SARS-CoV-infected animals versus aged PBS-infected macaques. The central node is NF-κB, a key factor in inflammation and development of ARDS. Genes depicted in green are downregulated and in red upregulated. As a reference, the same network is shown for young adult animals (left panel) and aged animals (right panel). (B–C) Gene expression profiles showing differentially expressed NF-κB target genes (B) and genes coding for proteins involved ARDS (C) of aged and young adult macaques. Gene sets were obtained from Ingenuity Pathways Knowledge Base or literature and changed ≥2-fold in at least one of the macaque groups as compared to PBS-infected controls. The data presented are error-weighted fold change averages for six young adult and aged animals. Genes shown in red were upregulated, in green downregulated, and in grey not significantly diferentially expressed in infected animals relative to PBS-infected animals (log (base 2) transformed expression values with minimum and maximum values of the color range being −4 and 4). See Table S2 and S3 for full gene names and expression values. (D) Lung sections of PBS and SARS-CoV-infected aged and young adult macaques were stained with an antibody against phosphorylated NF-κB (brown) and with a mouse-anti-SARS-nucleocapsid IgG2a (red). Sections were counterstained with hematoxylin. Original magnifications are ×40.

As NF-κB target genes were differentially regulated in the direct comparison of SARS-CoV-infected aged and young adult macaques (Figure 5), we focussed on NF-κB in the indirect comparison of aged and young adult SARS-CoV-infected macaques compared to aged and young adult PBS-infected animals, respectively. A gene interaction network, showing the interplay between “immune response”-type genes with NF-κB as central node, revealed that aged SARS-CoV-infected macaques showed a much more robust regulation of these genes than young adult infected animals (Figure 7A) compared to their respective PBS-infected animals, which was corroborated by an analysis of differentially expressed target genes of NF-κB (Figure 7B). Several of these genes, among which *VCAM1*, *F3*, *PTX3*, and *IL-8*, have also been implicated in development of ARDS (Figure 7C) [9],[27],[28].

In order to visualize NF-κB-signalling in the lungs of SARS-CoV-infected aged and young adult macaques, translocation of NF-κB was studied using immunohistochemistry with antibodies against phosphorylated NF-κB on day 4 after infection. As shown in Figure 7D, hardly any phosphorylated NF-κB could be detected in the nuclei of cells of PBS-infected macaques, while in the lungs of SARS-CoV-infected animals, cells with phosphorylated NF-κB in their nuclei were abundantly present. Phosphorylated NF-κB was detected primarily in the nuclei of non-infected cells (Figure 7D). No obvious differences in the translocation of NF-κB in the lungs of aged and young adult macaques were observed.

### Type I interferon-β mRNA level is negatively correlated with gross pathology

**Figure 8:**
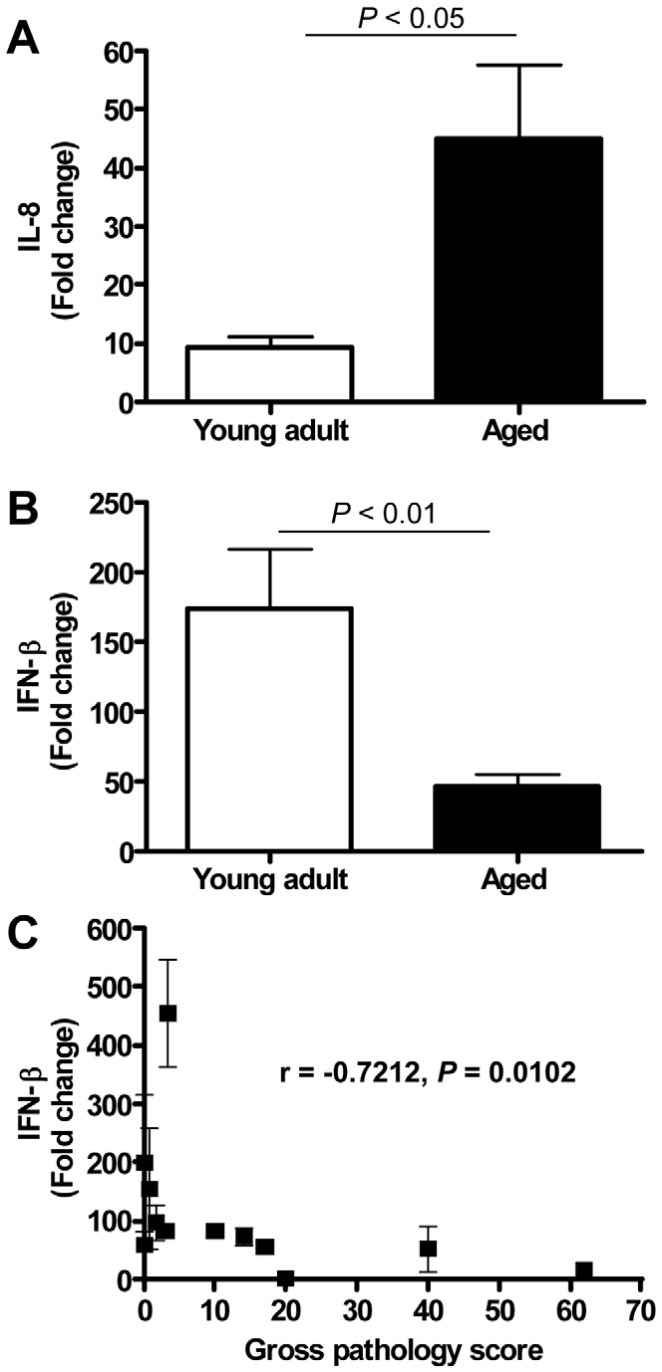
Quantitative RT-PCR confirmation of IFN-β mRNA levels. (A) Quantitative RT-PCR for IL-8 was performed on two-three separate lung samples per animal with substantial virus replication. The data presented are error-weighted (±s.e.m.) averages of the fold-change as compared to PBS-infected controls for young adult (*n* = 6) and aged (*n* = 6) animals. (B) Quantitative RT-PCR for IFN-β was performed on two-three separate lung samples per animal with substantial virus replication. The data presented are error-weighted (±s.e.m.) averages of the fold-change as compared to PBS-infected controls for young adult (*n* = 6) and aged (*n* = 6) animals. (C) The expression level of IFN-β (fold change) per animal was plotted against gross pathology score and the correlation coefficient was determined using Spearman's correlation test.

Overall, our data indicate that SARS-CoV-infected aged macaques display a stronger pro-inflammatory host response to infection than young adult macaques. For example, mRNA levels for IL8, a key player in ALI/ARDS and a potent chemotactic factor essential in acute inflammation that is induced by a wide range of stimuli among which IL1β, viral products, and oxidative stress, were strongly upregulated in SARS-CoV-infected aged macaques as compared to young adult animals (Figure 5B, 7B, 8A). Despite the overall stronger activation of innate host gene responses in SARS-CoV-infected aged animals, microarray analyses revealed that IFN-β, well-known for its antiviral activities, was not differentially expressed in aged macaques compared to PBS-infected animals, in contrast to young adults (Figure 6E). RT-PCR analysis confirmed differential expression of IFN-β mRNA between young adult and aged macaques (Figure 8B). As shown in Figure 3, this difference in IFN-β levels in aged and young adult macaques did not affect viral replication efficiency. IFN-β mRNA levels, however, negatively correlated with gross pathology (Figure 8C).

### Anti-inflammatory action of type I interferon mitigates pathology in SARS-CoV-infected aged macaques

**Figure 9:**
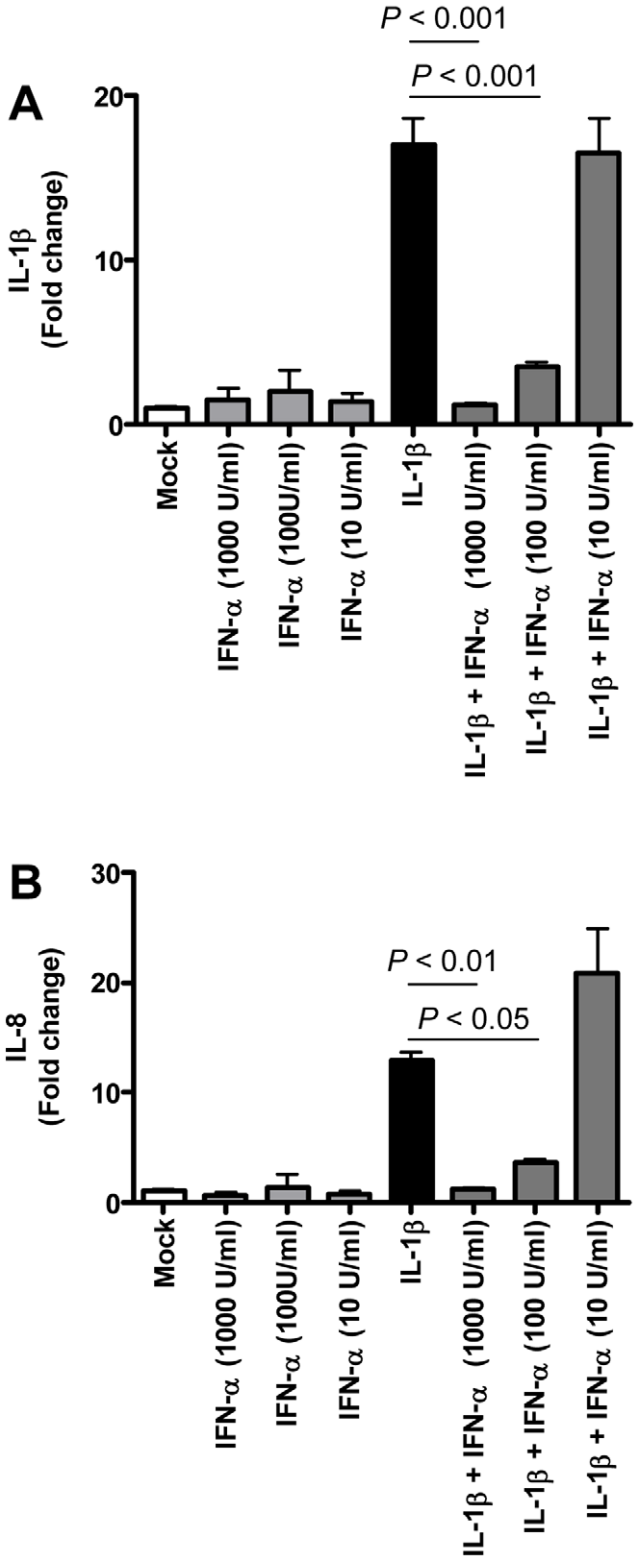
Anti-inflammatory type I IFN inhibits IL-1β-induced pro-inflammatory cytokine production in PBMCs. (A–B) Induction of IL-1β (A) and IL-8 (B) mRNAs after treatment of human PBMC with IL-1β (5 ng/ml), IFN-α (1000 U/ml, 100U/ml, or 10 U/ml) or both as determined by quantitative RT-PCR. The data presented are error-weighted (±s.e.m.) averages of the fold-change as compared to untreated (Mock) PBMC. Shown are representative data from one out of four donors.

The observation of a reverse correlation of IFN-β and IL-8 mRNA levels with age after SARS-CoV infection may reflect a physiological cross-regulation in which type I interferon and/or its respective signalling pathways modulate pro-inflammatory host responses [29],[30]. To corroborate this hypothesis, we treated uninfected human PBMC with IL-1β, which is known to rapidly activate NF-κB-signalling [31],[32], and observed the induction of pro-inflammatory cytokines in uninfected human PBMC, such as IL-1β and IL-8 (Figure 8A–B). An anti-inflammatory effect of pegylated IFN-α on IL-1β-induced responses was confirmed *in vitro*, as a dose-dependent inhibition of IFN-α on recombinant IL-1β-induced IL-1β and IL-8 mRNA levels in human PBMC was observed (Figure 9A–B).

**Figure 10:**
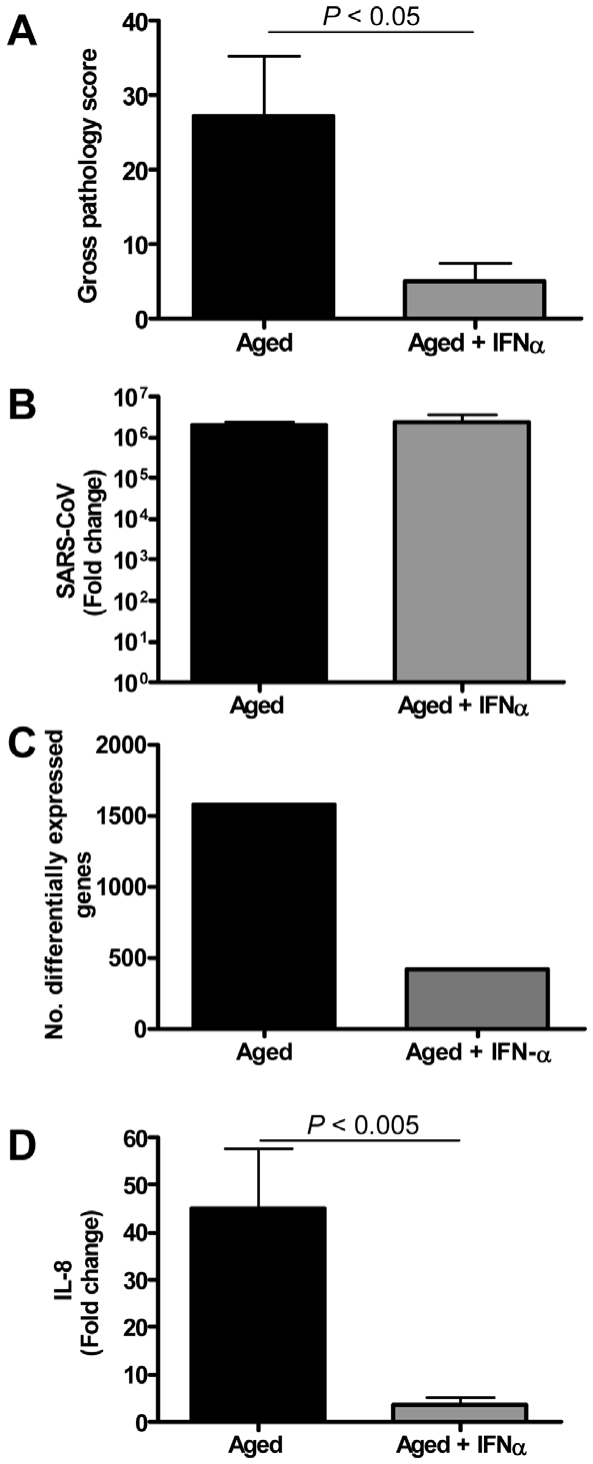
Anti-inflammatory type I IFN inhibits virus-induced ALI in aged SARS-CoV-infected macaques. (A) Gross pathology scores of lungs from macaques were determined during necropsy and averaged (±s.e.m.). (B) Average fold change (±s.e.m.) in SARS-CoV mRNA levels in the lungs of pegylated IFN-α-treated (*n* = 3) and untreated aged (*n* = 6) macaques compared to aged PBS-infected (*n* = 4) animals as determined by real-time RT-PCR. (C) Number of differentially expressed gene transcripts compared to aged PBS-infected animals (≥2-fold change). (D) Quantitative RT-PCR for IL-8 was performed on two-three separate lung samples per animal with substantial virus replication. The data presented are error-weighted (±s.e.m.) averages of the fold-change as compared to PBS-infected controls for aged animals (*n* = 6) and aged animals treated with IFN-α (*n* = 3).

Because type I IFNs can inhibit pro-inflammatory signalling pathways, among which NF-κB signalling pathways [29],[30], we examined whether exogenous administration of type I IFN in SARS-CoV-infected aged macaques could influence SARS-CoV pathogenesis. Retrospective analyses of the lungs of SARS-CoV-infected aged animals treated therapeutically with type I IFN [22] showed that SARS-CoV-infected IFN-treated aged animals remained free of clinical symptoms and had no or less extensive pulmonary consolidation than untreated aged macaques (Figure 10A). Virus titers in the lungs, however, were similar between IFN-treated and untreated aged macaques (Figure 10B) and viral antigen expression in the lungs was not significantly different [22].

In a direct comparison of the host response to infection in aged (*n* = 6) versus IFN-treated aged (*n* = 3) macaques using LIMMA, 961 gene transcripts were differentially expressed (fold change ≥2; *p*<0.05). Upon analysis of these gene transcripts within the context of genetic pathways, four of the most significantly regulated molecular/cellular functions (*p*<0.005) were associated with a pro-inflammatory response and included cellular growth and proliferation, cell death, cell movement, and cell-to-cell signalling, indicating that significant differences exist in the host response to SARS-CoV infection in animals treated with type I IFN compared to untreated aged macaques.

To obtain a broader view of the host response to infection, global gene expression profiles were determined in lungs of SARS-CoV-infected aged (*n* = 6) or IFN-treated aged macaques (*n* = 3) in comparison to aged PBS-infected macaques (*n* = 4). IFN-treated macaques differentially expressed (fold change ≥2; *p*<0.05) approximately four-fold less gene transcripts than untreated aged macaques (Figure 10C) as compared to PBS-infected animals. The most significantly regulated molecular/cellular functions in the IFN-treated macaque group compared to PBS-infected animals were associated with a pro-inflammatory response and included cellular growth and proliferation, cell death, cell movement, and cell-to-cell signalling, similar to what was observed for the aged macaque group, although less genes per function were differentially expressed (Figure S5A). These data suggested a common nature of the host response to infection in aged and IFN-treated aged animals, although the severity seemed different.

To gain more insight in differences in the host response to infection in aged and IFN-treated macaques compared to PBS-infected animals, heat maps were generated for differentially regulated genes involved in pro-inflammatory pathways apoptosis (Figure S5B) and cell adhesion (Figure S5C). Using Goeman's global test [26] on the defined gene subsets cell adhesion and apoptosis, significant differences between aged and IFN-treated animals in these pro-inflammatory pathways were obtained, providing statistical evidence for a difference in host response of aged and IFN-treated animals to SARS-CoV infection. Moreover, a decrease in differentially expressed target genes of NF-κB was observed (Figure S5D). Most notably, a dramatic decrease in the expression of cytokine/chemokine mRNA levels was observed, among which IL-8 (Figure 10D, Figure S5B–C). These data show that therapeutic treatment of SARS-CoV-infected aged macaques with type I IFN primarily results in downregulation of pro-inflammatory host responses.

## Discussion

### Age, pathology and pro-inflammatory host response go hand-in-hand

The present study aimed at gaining insight into the pathogenesis of SARS-CoV by studying the relationship between age, pathology, virus replication, and host response in a macaque model. In humans, SARS-CoV infection progresses from an atypical pneumonia to acute diffuse alveolar damage and ARDS [5]. The overall human fatality rate reached ∼10% and up to 50% in elderly [5],[6]. The acute lung injury observed after SARS-CoV infection in aged macaques is similar to what has been seen in humans that progress to ARDS [6]. This disease process includes an acute exudative phase, consisting of severe leukocyte infiltration, edema, the formation of hyaline membranes, and proliferation characterized by type II pneumocyte hyperplasia [33]. SARS-CoV-infected aged macaques develop more severe pathology than young adult animals, even though viral replication levels are similar. The chronic phase, which is characterized by persistent intra-alveolar and interstitial fibrosis and mortality was not observed because animals were sacrificed early after infection.

Comparative analyses of gene expression in aged and young adult SARS-CoV-infected macaques revealed that the host response to SARS-CoV infection is similar in nature, but differs significantly in severity in pro-inflammatory responses. Aged macaques had a stronger host response to virus infection than young adult macaques, with an increase in differential expression of genes associated with inflammation that center around the transcription factor NF-κB. Comparative analysis of PBS-infected aged and young adult macaques revealed significant differences in gene expression as a result of aging only. These observations are in line with earlier hypotheses that age-related accumulated oxidative damage and a weakened antioxidative defense system cause a disturbance in the redox balance, resulting in increased reactive oxygen species. Subsequently, the oxidative stress-induced redox imbalance activates redox-sensitive transcription factors, such as NF-κB, followed by the induction of pro-inflammatory genes including IL1β, IL6, TNFα and adhesion molecules, key players in the inflammatory process [34]. Oxidative stress may also potentiate the cellular responses to IL-1β [35], an early mediator of inflammation [36]. Thus, aging is associated not only with alterations in the adaptive immune responses, but also with a pro-inflammatory state in the host [34],[37],[38],[39]. Oxidative stress and toll-like receptor-4 signaling via NF-κB triggered by viral lung pathogens, such as SARS-CoV, may further amplify the host response ultimately resulting in ALI [25]. Taking the host gene expression profiles of PBS-infected aged and young adult macaques into account, we also observed a stronger activation of the pro-inflammatory pathways in SARS-CoV-infected aged macaques than in young adults. The finding that genes activated by NF-κB are significantly differentially upregulated in aged macaques infected with SARS-CoV is in line with the role of NF-κB as a redox-sensitive transcription factor in pro-inflammatory host responses and the development of ALI/ARDS [24],[25]. Given the fact that several SARS-CoV proteins block NF-κB signaling [40],[41],[42], we hypothesize that NF-κB-signaling in non-infected cells is largely responsible for the upregulated expression of NF-κB target genes, such as IL8, in aged compared to young adult macaques.

These observations are largely in line with transcriptome analyses in mice and SARS patients. In severe SARS patients, cytokines/chemokine involvement as the illness progresses may lead to widespread immune dysregulation and serious pathogenic events [13]. Aged mice show more pathology than young adult mice and the transcriptional profile in aged mice generally indicates a more robust pro-inflammatory response to virus infection than in young mice [43],[44].

### Type I IFN signalling

Previously, we demonstrated IFN induction and signalling in SARS-CoV-infected macaques early after infection [19]. Based on the observation that plasmacytoid dendritic cells are able to produce type I IFNs after SARS-CoV infection in vitro [45], it was speculated that these cells are the IFN-producing cells in lungs of SARS-CoV-infected macaques. In addition, phosphorylated STAT-1 was observed in the nuclei of numerous cells in the lungs of SARS-CoV-infected macaques, indicating that these cells had been activated by IFNs or other agonists produced in the lung [19]. In SARS-CoV-infected cells, however, STAT-1 signalling was blocked [19], consistent with the fact that a range of SARS-CoV proteins can function as interferon antagonists that inhibit IFN production and signalling [41],[42]. Therefore, a large part of the genes activated downstream of STAT-1, observed in genomics analyses, is likely due to signalling in non-infected cells [19]. In the current study, we observed that aged macaques expressed significantly lower levels of IFN-β mRNA than young adult macaques and that IFN-β mRNA levels correlated negatively with severity of pathology. Interestingly, aged and young adult SARS-CoV-infected macaques showed opposite expression patterns for type I IFN-β and certain pro-inflammatory cytokines, such as IL-8. These data are corroborated by previous observations showing that higher amounts of pro-inflammatory cytokines, such as IL-1β and IL-8, are produced upon stimulation of leukocytes of the elderly, whereas induction of type I IFNs is decreased compared to young adults [46],[47],[48].

### Cross-regulation between type I IFN and NF-κB signalling cascades

The observation of a reverse correlation of IFN-β and IL-8 mRNA levels with age after SARS-CoV infection may reflect a physiological cross-regulation between antiviral STAT-1 and proinflammatory NF-κB pathways. Evidence for such a cross-regulation between type I IFN/STAT-1 and pro-inflammatory/NF-κB signaling pathways exists. Type I interferons exert significant anti-inflammatory effects and provide at least partial protection from disease in collagen-induced arthritis, auto-immune encephalitis, and multiple sclerosis [49],[50],[51],[52],[53]. Not only inhibits IFN-beta expression of the IL8 gene at the transcriptional level [54], type I IFNs can also activate TAM receptor tyrosine kinases that inhibit toll-like receptor-induced cytokine-receptor cascades [55],[56] and induce the immunosuppresive cytokine IL-10 [57]. Direct NF-κB/STAT-1 protein-protein interactions [58] and modification of STAT-1 by acetylation, may be involved in this process [30]. A loss of type I IFN/STAT-1 signaling in aged macaques may negatively regulate interferon-induced gene expression and type I IFN signaling, which may lead to enhanced inflammatory responses. On the other hand, increased activation of NF-κB signaling pathways in aged macaques may negatively regulate interferon-induced gene expression and type I IFN signaling [29],[59],[60], which may enhance pro-inflammatory responses even further.

**Figure 11:**
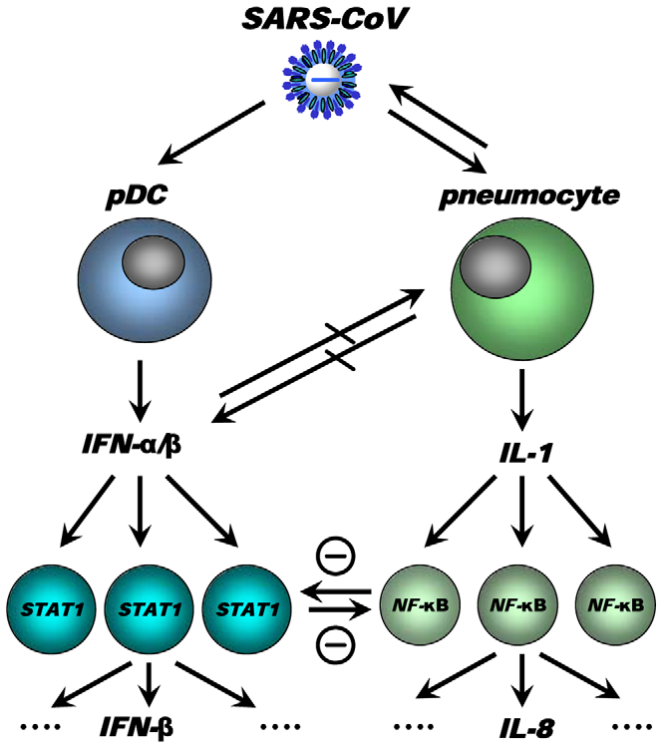
Model for cross-talk between “pro-inflammatory” and “antiviral” pathways during SARS-CoV infection. SARS-CoV infection results in activation of both “antiviral” and “pro-inflammatory” pathways. Subsets of uninfected cells, depicted by pDCs, start producing type I IFN (IFN-α), which results in STAT-1 activation in neighbouring cells, which in turn may produce other mediators (e.g. IFN-β). The SARS-CoV-infected cells produce inflammatory mediators, supposedly IL-1, which results in NF-κB activation in neighbouring uninfected cells and subsequent production of inflammatory mediators, such as IL-8. Cross-regulation between “antiviral” and “pro-inflammatory” pathways allows polarisation of antiviral or pro-inflammatory responses thereby modulating pathology. Modulation of transcription factors in the uninfected cells, e.g. by aging, may affect the overall outcome of the infection.

We have integrated our data and other findings on cross-regulation in a model (Figure 11). The model depicts the innate immune response to SARS-CoV infection as a coordinated series of signaling pathways aimed at clearing the virus while not harming host cells. Upon SARS-CoV infection, infected cells, depicted in the model as pneumocytes, produce inflammatory mediators that activate NF-κB, resulting in the production of pro-inflammatory cytokines and chemokines, such as IL-8. IL-1 is one of the cytokines highly upregulated on day 1 after infection upon SARS-CoV infection of macaques [19] and capable of activating NF-κB. At the same time, the virus is recognized by sentinel cells, such as pDCs, that produce type I IFNs to signal that a foreign invader has entered the host. The production of IFN induces neighboring non-infected cells to remodel the intracellular environment by producing a range of antiviral proteins, aiding in a block of viral replication. A cross-regulation between the “antiviral” and “pro-inflammatory” pathways occurs, which is a critical requirement to allow fine-tuning of the host response to infection and return to homeostasis. Disease outcome may be determined by the relative contribution of “antiviral” and “pro-inflammatory” pathways and apparently aging influences this intricate balance significantly.

Causal relationships between “antiviral” and “pro-inflammatory” pathways in macaques are difficult to prove and future studies in specific gene knock-out mice should therefore further clarify the complex interactions in the response to SARS-CoV. Our own *in vitro* experiments and the type I IFN intervention in SARS-CoV-infected aged macaques indicate that type I IFNs can play a role in mitigating pro-inflammatory host responses and severity of pathology. Therapeutic treatment of SARS-CoV-infected aged macaques with type I IFN reduces pathology and diminishes pro-inflammatory gene expression, including IL-8 levels, without affecting virus replication in the lungs. Antiviral effects of type I IFNs were not obvious, probably due to the fact that SARS-CoV infected cells inhibit STAT-1 signalling and viral replication peaks early after infection when treatment with pegylated IFN-α started. Given the fact that phosphorylated NF-κB was present mainly in the nuclei of non-infected cells in the lungs of SARS-CoV-infected macaques, these cells are potential targets for the action of IFN and subsequent STAT-1 signalling. It remains uncertain whether endogenously produced IFNs in young adult macaques are essential in the control of inflammatory responses or that enhanced activation of inflammatory pathways simply does not occur. Our data are in line with the observation that treatment of SARS-CoV-infected aged mice with type II IFN-γ, which like type I IFN also signals via STAT-1, protected against lethal respiratory illness, seemingly without an effect on viral replication [61]. Moreover, in humans with SARS, use of type I IFNs was associated with reduced disease-associated hypoxia and a more rapid resolution of radiographic lung abnormalities [62]. Whether the anti-inflammatory action of type I/II IFNs in macaques, mice and humans occurs via common pathways and is interchangeable between host species remains to be determined. Assuming that there is a conserved pathway in ALI/ARDS induced by multiple pathogens, including pandemic viruses that may emerge from avian influenza, modulation of the host response by type I IFNs provides a promising outlook for novel intervention strategies.

## Materials and Methods

### Macaque studies

Six young adult cynomolgus macaques (*Macaca fascicularis*), 3–5 years old, four of which carried active temperature transponders in the peritoneal cavity, and four aged cynomolgus macaques, 10–18 years old, which all carried active temperature transponders, were inoculated with SARS-CoV strain HKU39849, as described previously [19],[21],[22],[23]. Two additional aged animals (17 and 19 years old), previously infected with SARS-CoV strain HKU39849 [22], were enrolled in this study as well. Four young adult mock (PBS) infected animals from a previous study [19] and four aged macaques were taken as controls. Lung tissues stored in RNA-later from three cynomolgus macaques, 13 years old, previously inoculated with SARS-CoV strain HKU39849 and treated with pegylated IFN-α at a dose of 3 µg/kg intramuscularly on days 1 and 3 after infection, were taken along for molecular analyses [22]. All animals were infected with the same dose of virus, using the same inoculation procedure, by the same person to minimize inter-experiment variation. All animals were checked daily for clinical signs and anaesthetised with ketamine on days 0, 2 and 4 after infection to collect oral, nasal, and rectal swabs [22]. All animals were euthanized on day 4 post infection. Necropsies and sampling for histology/immunohistochemistry were performed as described [22]. The percentage of affected lung tissue from each lung lobe was determined at necropsy, recorded on a schematic diagram of the lung and the area of affected lung tissue was subsequently calculated (gross pathology score).

### Ethics

Approval for animal experiments was obtained from the Institutional Animal Welfare Committee and performed according to Dutch guidelines for animal experimentation.

### Immunohistochemistry

Serial 3 µm lung sections were stained using mouse-anti-SARS-nucleocapsid IgG2a (clone Ncap4; Imgenex) 1∶1600, mouse-anti-human neutrophil elastase (clone NP-57; DAKO) 1∶10, mouse-anti-human CD68 (clone KP1; DAKO) 1∶200, mouse-anti-human pankeratin (clone AE1/AE3; Neomarkers) 1∶100, rabbit anti p-NF-κB p65 (Santa Cruz) or rabbit control and isotype antibodies (clones 11711 and 20102; R&D), according to standard protocols [22],[23]. Quantitative assessment of SARS-CoV infection in the lungs was performed as described previously [22].

### RNA-extraction and quantitative RT-PCR

RNA from 200 µl of swabs was isolated with the Magnapure LC total nucleic acid isolation kit (Roche) external lysis protocol and eluted in 100 µl. SARS-CoV RNA was quantified on the ABI prism 7700, with use of the Taqman Reverse Transcription Reagents and Taqman PCR Core Reagent kit (Applied Biosystems), using 20 µl isolated RNA, 1× Taqman buffer, 5.5 mM MgCl_2_, 1.2 mM dNTPs, 0.25 U Amplitaq gold DNA polymerase, 0.25 U Multiscribe reverse transcriptase, 0.4 U RNAse-inhibitor, 200 nM primers, and 100 nM probe [23]. Amplification parameters were 30 min at 48°C, 10 min at 95°C, and 40 cycles of 15 s at 95°C, and 1 min at 60°C. RNA dilutions isolated from a SARS-CoV stock were used as a standard. Average results (±s.e.m.) for young adult (*n* = 6) and aged macaque (*n* = 4) groups were expressed as SARS-CoV equivalents per ml swab medium.

Lung tissue samples (0.3–0.5 gram) were taken for RT-PCR and microarray analysis in RNA-later (Ambion, Inc.). RNA was isolated from homogenized post mortem tissue samples using Trizol Reagent (Invitrogen) and the RNeasy mini kit (Qiagen). cDNA synthesis was performed with 1 µg total RNA and Superscript III RT (Invitrogen) with oligo(dT), according to the manufacturer's instructions. Semi-quantitative RT-PCR was performed to detect SARS-CoV mRNA and to validate cellular gene expression changes as detected with microarrays [19]. Differences in gene expression are represented as the fold change in gene expression relative to a calibrator and normalized to a reference, using the 2^−ΔΔCt^ method [63]. GAPDH (glyceraldehydes-3-phosphate dehydrogenase) was used as endogenous control to normalize quantification of the target gene. The samples from the young adult PBS-infected macaques were used as a calibrator. Average results (±s.e.m.) for young adult (*n* = 6), aged (*n* = 6), and IFN-α-treated aged (*n* = 3) macaque groups were expressed as fold change compared to young adult PBS-infected animals, respectively [63]. In addition, groups were based on severity of pathology: young adult macaques (*n* = 6), aged macaques with pathology (*n* = 4), and aged macaques with severe pathology with >40% of lungs affected (*n* = 2) (Supplementary Figure 4). As titration of lung homogenates gave inconsistent results in our hands and because the effects of endogenous and exogenous IFN may influence titration outcomes, we chose taqman and immunohistochemistry to determine viral replication levels in the lung.

### Isolation and activation of PBMC

PBMC from healthy blood donors were isolated from heparinized venous blood using Lymphoprep (Axis-Shield). PBMC were resuspended at 2×10^6^/ml in RPMI 1640 medium (Biowhittaker) supplemented with L-glutamine (2 mM), penicillin (100 U/ml), streptomycin (100 µg/ml), and 10% fetal calf serum. Freshly isolated PBMC were incubated with IL-1β (5 ng/ml; eBioscience), IFN-α 2a (1000 U/ml, 100U/ml, or 10U/ml; Roferon-A; Roche) or both for 24 hours in duplo or triplo per donor. Total RNA from stimulated PBMC was isolated using Trizol Reagent (Invitrogen) and the RNeasy mini kit (Qiagen). cDNA synthesis was performed with 100 ng total RNA and Superscript III RT (Invitrogen) with oligo(dT), according to the manufacturer's instructions. Semi-quantitative RT-PCR was performed for IL-8 [19] and IL-1β (Taqman gene expression assays; Applied Biosystems) as described previously using the 2^−ΔΔCt^ method [63]. Average results (±s.e.m.) were expressed as fold change compared to untreated (mock) cells [63].

### Statistical analysis

Data (RT-PCR and gross pathology scores for SARS-CoV-infected young adult versus aged and aged versus aged animals treated with IFN) were compared using Student's t-test with Welch's correction. Differences were considered significant at *P*<0.05. One-way ANOVA and Bonferroni's multiple comparison test were used for the comparison of data in groups based on severity of pathology (low, medium, high) and *in vitro* IFN inhibition experiments. Correlation coefficients were determined using Spearman's correlation test.

### RNA labeling, microarray hybridization, scanning and data preprocessing

Pooled total RNA (2.4 µg) from one-three separate lung pieces of all animals (including previously infected animals), with substantial SARS-CoV replication (>10^5^ fold change), was labeled using the One-Cycle Target Labeling Assay (Affymetrix) and hybridized onto Affymetrix GeneChip Rhesus Macaque Genome Arrays (Affymetrix), according to the manufacturer's recommendations. Image analysis was performed using Gene Chip Operating Software (Affymetrix). Microarray Suite version 5.0 software (Affymetrix) was used to generate .dat and .cel files for each experiment. All data were normalized using a variance stabilization algorithm (VSN) [64]. Transformed probe values were summarized into one value per probe set by the median polish method [65]. Primary data is available at http://www.virgo.nl in accordance with proposed MIAME standards.

### Microarray data analysis

Probe set (gene) wise comparisons between the experimental conditions (aged, young adult and IFN-treated animals versus young adult or aged PBS-infected animals and directly compared to each other) were performed by LIMMA (version 2.12.0) [66]. Correction for multiple testing was achieved by requiring a false discovery rate (FDR) of 0.05, calculated with the Benjamini-Hochberg procedure [67]. To understand the gene functions and the biological processes represented in the data and obtain differentially expressed molecular and cellular functions, Ingenuity Pathways Knowledge Base (http://www.ingenuity.com/) was used. Heat maps of pro-inflammatory pathways were produced using complete linkage and Euclidian distance in Spotfire DecisionSite for Functional Genomics version 9.1 (http://www.spotfire.com/) and Ingenuity Pathways Knowledge Base (http://www.ingenuity.com/), using log (base 2) transformed expression values with minimum and maximum values of the color range being −4 and 4, respectively. Differences between conditions in expression of specific pro-inflammatory pathways, e.g. direct comparison of defined gene sets (aged versus young adult and aged versus aged IFN-treated animals), were tested by Goeman's global test procedure [26]. Hierarchical clustering analysis of normalized log-2 based hybridization signals of individual young adult and aged macaques of a set of gene transcripts that were identified as being differentially regulated (fold change ≥2; FDR<0.05) in at least one of the comparisons of young adult versus young adult PBS or aged versus aged PBS animals were created using Spotfire DecisionSite for Functional Genomics version 9.1 (http://www.spotfire.com/) with complete linkage and Eucledian distance parameters.

## Supporting Information

Table S1 Annotated differentially expressed genes in aged versus young adult SARS-CoV infected macaques.
(0.15 MB DOC)

Table S2 Description of genes
(0.04 MB DOC)

Table S3 Log (base 2)-transformed expression values of genes in the heat maps in Fig 6, Fig. 7, and Fig. S5.
(0.03 MB DOC)

Table S4 Log (base 2)-transformed expression values of genes in heat maps in Fig. S4
(0.03 MB DOC)

Figure S1 Gross lesions in aged macaque. (A) SARS-CoV-induced lesions (white arrows) in the lung are still visible after inflation with 10% neutral-buffered formalin. (B–C) Schematic diagrams of the lungs showing gross pathology lesions of SARS-CoV-infected young adult (B) and aged (C) macaques.
(0.31 MB PDF)

Figure S2 Histology and immunohistochemical detection of cells in lungs from SARS-CoV-infected macaques. (A–B) Lesion in the lung of a SARS-CoV-infected aged macaque, characterized by thickened alveolar walls lined by type II pneumocytes (type II pneumocyte hyperplasia) with influx of inflammatory cells. Consecutive sections were stained with a mouse monoclonal anti-human CD68 antibody for macrophages (A) and a mouse monoclonal anti-human neutrophil elastase antibody for neutrophils (B). Sections were counterstained with hematoxylin. (C) Lesions in the lung of a SARS-CoV infected aged macaque showing diffuse alveolar damage, characterized by type II pneumocyte hyperplasia with influx of inflammatory cells. (D) Lesion in the lung of a SARS-CoV-infected aged macaque, characterized by thickened alveolar walls lined by type II pneumocytes stained with a mouse monoclonal anti-human pankeratin antibody for epithelial cells. (E–F) Hyaline membranes (E) and syncytia (F) were occasionally observed in the lungs of aged macaques. Original magnifications are ×20 and ×40.
(0.11 MB PDF)

Figure S3 Global gene expression profiles of individual young adult and aged animals. For a subset of gene transcripts, cytokines and chemokines, normalized log-2 based hybridization values for individual aged and young adult macaques are shown.
(0.01 MB PDF)

Figure S4 Microarray analyses of the lower respiratory tract of SARS-CoV-infected macaques displaying different levels of severity of pathology. (A) Gross pathology scores of the lungs from aged and young adult macaques were determined. Based on the severity of pathology, macaques were divided in three groups (low (young adult; n = 6), medium (aged; n = 4), and high (aged; n = 2) pathology score), and average pathology scores (±s.e.m.) are shown. (B) Number of differentially expressed gene transcripts compared to uninfected animals (≥2-fold change) in macaque groups. (C) Number of differentially expressed genes in macaque groups compared to PBS-infected animals with functions in cellular growth and proliferation, cell movement, cell death, or cell-to-cell signaling and interaction obtained from Ingenuity Pathways Knowledge Base. (D) Average fold change (±s.e.m.) in SARS-CoV mRNA levels in the lungs of macaques with low, medium and high pathology scores as compared to PBS-infected animals as determined by real-time RT-PCR. (E–G) Gene expression profiles showing differentially expressed genes coding for proteins involved in cell adhesion (E), proteins involved in apoptosis (F), and cytokines and chemokines (G) of macaque groups with low, medium and high pathology scores as compared to PBS-infected animals. Genes displayed were obtained from Ingenuity Pathways Knowledge Base and changed ≥2-fold in at least one of the macaque groups as compared to PBS-infected controls. The data presented are error-weighted averages. Genes shown in red were upregulated and in green downregulated in infected animals relative to PBS-infected animals (log (base 2) transformed expression values with minimim and maximum values of the color range being −4 and 4). Genes shown in grey were not significantly differentially regulated. See Table S2 and S4 for full gene names and expression values.
(0.05 MB PDF)

Figure S5 Microarray analyses of the lower respiratory tract of SARS-CoV-infected aged and aged macaques treated with pegylated IFN-α. (A) Number of differentially expressed genes in macaque groups compared to PBS-infected animals with functions in cell growth and proliferation, cell movement, cell death, or cell-to-cell signaling and interaction obtained from Ingenuity Pathways Knowledge Base. When SARS-CoV-infected aged macaques were compared directly to IFN-treated aged macaques, these gene sets were significantly differentially expressed. (B–D) Gene expression profiles showing differentially expressed genes coding for proteins involved in apoptosis (B), cell adhesion (C), or NF-κB-signaling (D) of IFN-α-treated and untreated aged macaques. Genes displayed were obtained from Ingenuity Pathways Knowledge Base or literature and changed ≥2-fold in at least one of the groups as compared to PBS-infected controls. The data presented are error-weighted averages. Genes shown in red were upregulated, in green downregulated, and in grey not significantly differentially expressed in infected animals relative to PBS-infected animals (log (base 2) transformed expression values with minimum and maximum values of the color range being −4 and 4). Global test analysis of the direct contrast of SARS-CoV-infected aged versus IFN-treated aged animals showed that the cell adhesion and apoptosis pathways were significantly differentially expressed (p<0.05). See Table S2 and S3 for full gene names and expression values.
(0.03 MB PDF)
