# Supplementary material for: Exacerbated Innate Host Response to SARS-CoV in Aged Non-Human Primates
Source: PLoS Pathog. 2010 Feb 5;6(2):e1000756. doi: 10.1371/journal.ppat.1000756 (PMC2816697; doi:10.1371/journal.ppat.1000756)
Supplement: Table S1 — Annotated differentially expressed genes in aged versus young adult SARS-CoV infected macaques. (0.15 MB DOC) [file ppat.1000756.s001.doc]

##### Supplementary Table S1 Annotated differentially expressed genes in aged versus young adult SARS-CoV infected macaques.
