# Supplementary material for: Exacerbated Innate Host Response to SARS-CoV in Aged Non-Human Primates
Source: PLoS Pathog. 2010 Feb 5;6(2):e1000756. doi: 10.1371/journal.ppat.1000756 (PMC2816697; doi:10.1371/journal.ppat.1000756)
Supplement: Table S2 — Description of genes (0.04 MB DOC) [file ppat.1000756.s002.doc]

##### Supplementary Table S2 Description of genes

**Gene name Gene description**

ADAM9 ADAM metallopeptidase domain 9

ANGPT1 Angiopoietin 1

APOE Apolipoprotein E

BAK1 Bcl2-homologous antagonist/killer

BGN Biglycan

BMP1 Bone morphogenetic protein 1

BTG2 B-cell translocation gene 2

CAMP Cathelicidin antimicrobial peptide

CASP4 Caspase 4

CCL11 Chemokine (C-C motif) ligand 11

CCL19 Chemokine (C-C motif) ligand 19

CCL25 Chemokine (C-C motif) ligand25

CCL3 Chemokine (C-C motif) ligand 3

CCL4L1 Chemokine (C-C motif) ligand 4 like 1

CCL8 Chemokine (C-C motif) ligand 8

CCNB1 Cyclin B1

CCR1 Chemokine (C-C motif) receptor 1

CCR5 Chemokine (C-C motif) receptor 5

CD47 CD47 antigen

CDH13 Cadherin 13

CFB Complement factor B

CST6 Cytostatin 6

CTNND1 Catenin, delta 1

CXCL1 Chemokine (C-X-C motif) ligand 1

CXCL1 /// CXCL3 Chemokine CXCL1///CXCL3

CXCL10 Chemokine (C-X-C motif) ligand 10

CXCL11 Chemokine (C-X-C motif) ligand 11

CXCL6 Chemokine (C-X-C motif) ligand 6

CXCL9 Chemokine (C-X-C motif) ligand 9

DKK3 Dickkopf homolog 3

EDN1 Endothelin 1

EGFR Epidermal growth factor receptor

EPAS1 Endothelial PAS domain protein 1

EPHA2 Ephrin receptor A2

F11R F11 receptor

F3 Coagulation factor III

FBLN5 Fibulin 5

FGF2 Heparin-binding growth factor 2

FOXP1 Forkhead box F1

FUT3 Fucosyltransferase 3

GADD45A Growth arrest & DNA-damage-inducible protein 45α

GZMA Granzyme A

GZMB Granzyme B

IFNB1 Interferon, beta

IGF1 Insulin-like growth factor 1

IKBKB Inhibitor κ light polypeptide gene enhancer B-cells kinase beta

IL15RA Interleukin 15 receptor, alpha

IL1RN Interleukin 1 receptor antagonist

IL2 Interleukin 2

IL29 Interleukin 29

IL6 Interleukin 6

IL8 Interleukin 8

IRF1 Interferon regulatory factor 1

IRF7 Interferon regulatory factor 7

ITGA6 Integrin A6

ITPR1 Inositol 1,4,5-triphosphate receptor, type 1

KIT V-kit Hardy-Zucker man4 feline sarcoma viral oncogene homolog

**Gene name Gene description**

LAMB1 Laminin, beta 1

LAMC1 Laminin, gamma 1

LGALS3BP Galectin 3 binding protein

LMNB1 Lamin B1

MCP-1 Chemokine (C-C motif) ligand 2

MET Met proto-oncogene tyrosine kinase

MMP9 Matrix metallopeptidase 9

MYBL1 V-myb myeloblastosis viral oncogene homolog-like 1

MYBL2 V-myb myeloblastosis viral oncogene homolog-like 2

MYC Myc proto-oncogene protein

NEO1 Neogenin homolog 1

NP Nucleoside phosphorylase

PCDHA11 Protocadherin 11

PCDHA5 Protocadherin 5

PIK3R1 Phosphoinositide-3-kinase, regulatory subunit 1

PLA2G2A Phospholipase A2, group IIA

PPBP Chemokine (C-X-C motif) ligand 7

PTPRB Protein tyrosine phosphatase, receptor type, B

PTPRF Protein tyrosine phosphatase, receptor type, F

PTX3 Pentraxin 3

RAD23B UV excision repair protein RAD23B homlog B

S100A8 S100 calcium-binding protein A8

SERPINA1 Alpha-1-antitrypsin

SERPING1 Serpin peptidase inhibitor 1 G1

SFTPD Surfactant protein D

SLIT2 Slit homolog 2

SOCS1 Suppressor of cytokine signaling 1

SPP1 Osteopontin

STAT1 Signal transducer and activator of transcription 1

TAPBP TAP-binding protein

TFPI2 Tissue factor pathway inhibitor 2

THBD Thrombomodulin

THBS3 Thrombospondin 3

TIMP3 Tissue inhibitpr of matrix metalloproteinase-3

TMOD1 Tropomodulin 1

TNFAIP3 Tumor necrosis factor, alpha-induced protein 3

TNFRSF17 Tumor necrosis factor receptor superfamily, member 17

TNFRSF19 Tumor necrosis factor receptor superfamily, member 19

TNFRSF21 Tumor necrosis factor receptor superfamily, member 21

TNFSF13B Tumor necrosis factor superfamily, member 13B

TOP2A DNA topoisomerase II, alpha isozyme

TSTA3 Tissue specific transplantation antigen P35B

VCAM1 Vascular cell adhesion molecule 1
