# Supplementary material for: Exacerbated Innate Host Response to SARS-CoV in Aged Non-Human Primates
Source: PLoS Pathog. 2010 Feb 5;6(2):e1000756. doi: 10.1371/journal.ppat.1000756 (PMC2816697; doi:10.1371/journal.ppat.1000756)
Supplement: Table S3 — Log (base 2)-transformed expression values of genes in the heat maps in Fig 6, Fig. 7, and Fig. S5. (0.03 MB DOC) [file ppat.1000756.s003.doc]

##### Supplementary Table S3 Log (base 2)-transformed expression values of genes in heat maps in Fig 6, Fig. 7, and Fig. S5.
