# Supplementary figures and images for: Exacerbated Innate Host Response to SARS-CoV in Aged Non-Human Primates
Source: PLoS Pathog. 2010 Feb 5;6(2):e1000756. doi: 10.1371/journal.ppat.1000756 (PMC2816697; doi:10.1371/journal.ppat.1000756)

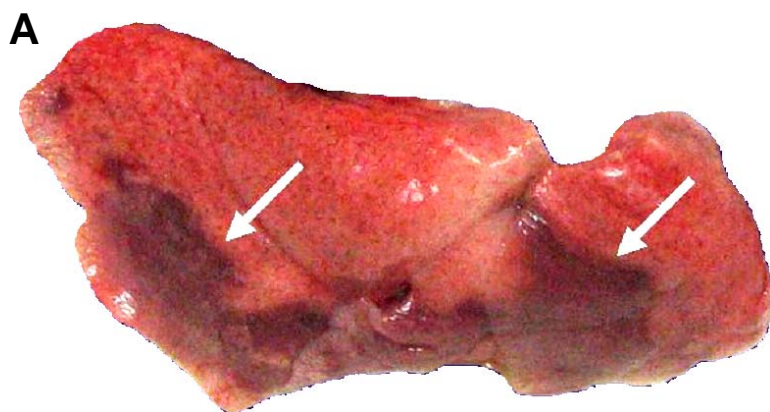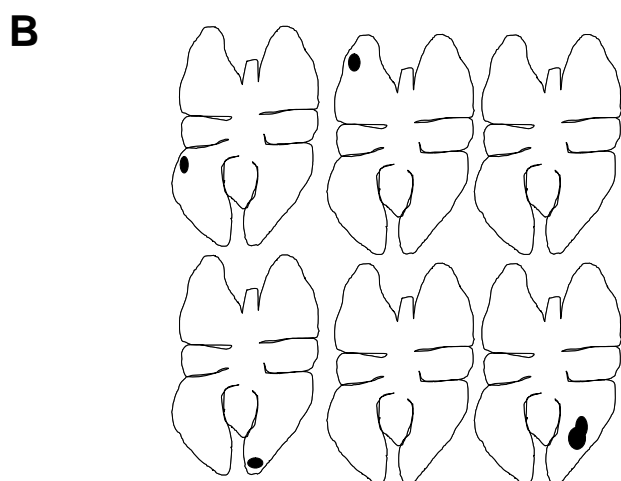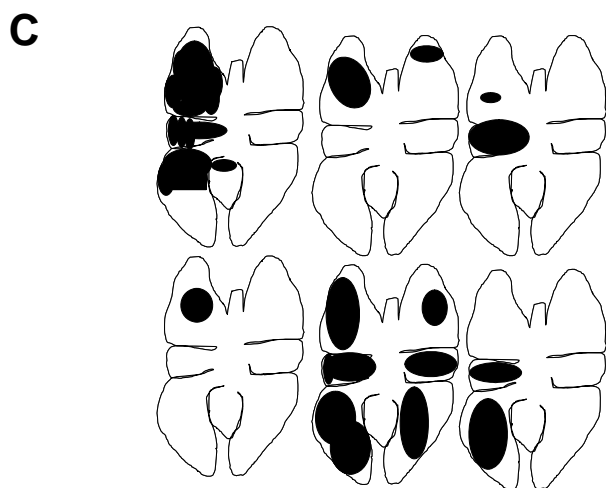

**Fig. S1**

Supplement: Figure S1 — Gross lesions in aged macaque. (A) SARS-CoV-induced lesions (white arrows) in the lung are still visible after inflation with 10% neutral-buffered formalin. (B–C) Schematic diagrams of the lungs showing gross pathology lesions of SARS-CoV-infected young adult (B) and aged (C) macaques. (0.31 MB PDF) [file ppat.1000756.s005.pdf]

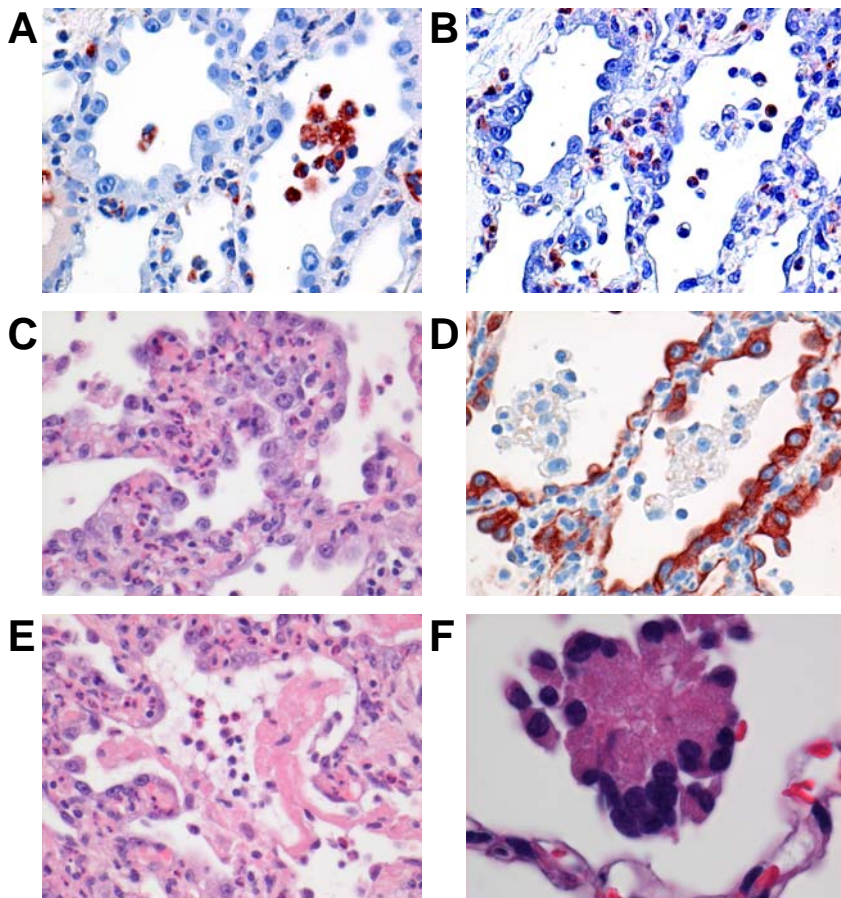

**Fig. S2**

Supplement: Figure S2 — Histology and immunohistochemical detection of cells in lungs from SARS-CoV-infected macaques. (A–B) Lesion in the lung of a SARS-CoV-infected aged macaque, characterized by thickened alveolar walls lined by type II pneumocytes (type II pneumocyte hyperplasia) with influx of inflammatory cells. Consecutive sections were stained with a mouse monoclonal anti-human CD68 antibody for macrophages (A) and a mouse monoclonal anti-human neutrophil elastase antibody for neutrophils (B). Sections were counterstained with hematoxylin. (C) Lesions in the lung of a SARS-CoV infected aged macaque showing diffuse alveolar damage, characterized by type II pneumocyte hyperplasia with influx of inflammatory cells. (D) Lesion in the lung of a SARS-CoV-infected aged macaque, characterized by thickened alveolar walls lined by type II pneumocytes stained with a mouse monoclonal anti-human pankeratin antibody for epithelial cells. (E–F) Hyaline membranes (E) and syncytia (F) were occasionally observed in the lungs of aged macaques. Original magnifications are ×20 and ×40. (0.11 MB PDF) [file ppat.1000756.s006.pdf]

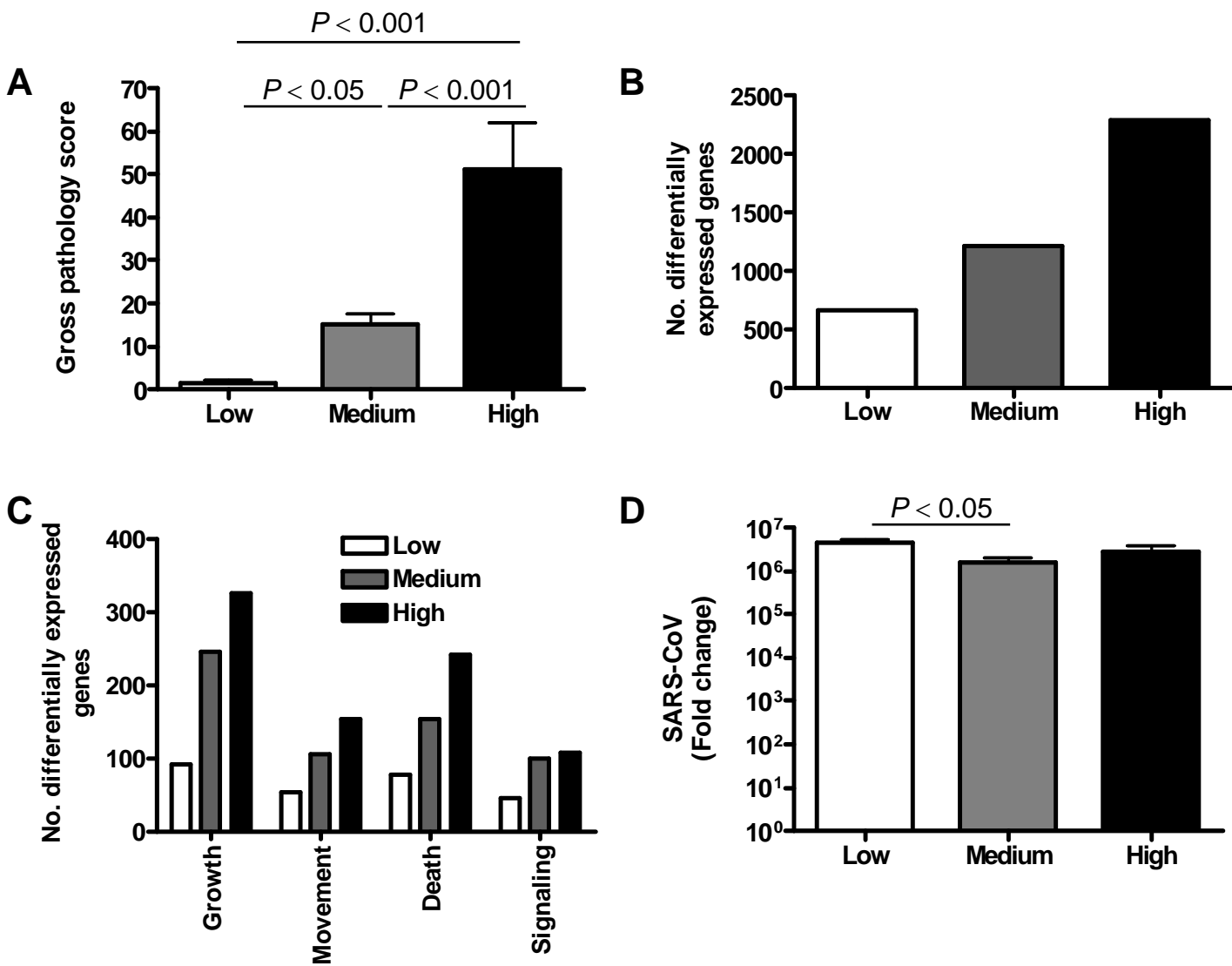

Fig. S4

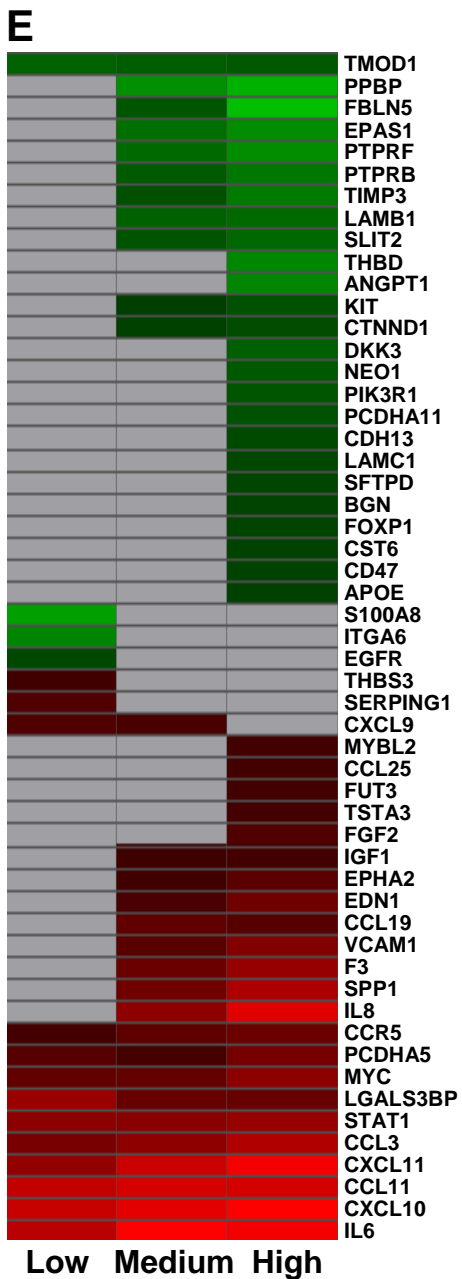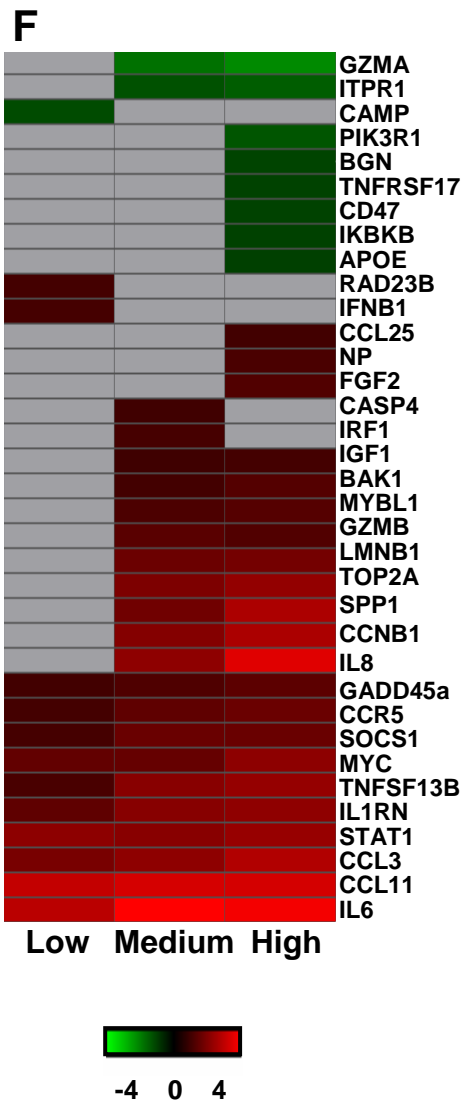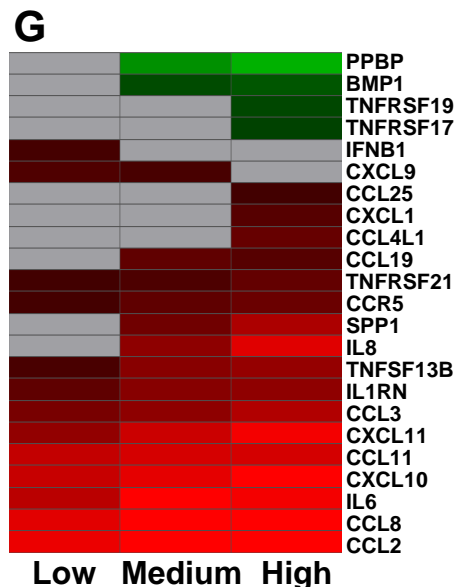

**Fig. S4**

Supplement: Figure S4 — Microarray analyses of the lower respiratory tract of SARS-CoV-infected macaques displaying different levels of severity of pathology. (A) Gross pathology scores of the lungs from aged and young adult macaques were determined. Based on the severity of pathology, macaques were divided in three groups (low (young adult; n = 6), medium (aged; n = 4), and high (aged; n = 2) pathology score), and average pathology scores (±s.e.m.) are shown. (B) Number of differentially expressed gene transcripts compared to uninfected animals (≥2-fold change) in macaque groups. (C) Number of differentially expressed genes in macaque groups compared to PBS-infected animals with functions in cellular growth and proliferation, cell movement, cell death, or cell-to-cell signaling and interaction obtained from Ingenuity Pathways Knowledge Base. (D) Average fold change (±s.e.m.) in SARS-CoV mRNA levels in the lungs of macaques with low, medium and high pathology scores as compared to PBS-infected animals as determined by real-time RT-PCR. (E–G) Gene expression profiles showing differentially expressed genes coding for proteins involved in cell adhesion (E), proteins involved in apoptosis (F), and cytokines and chemokines (G) of macaque groups with low, medium and high pathology scores as compared to PBS-infected animals. Genes displayed were obtained from Ingenuity Pathways Knowledge Base and changed ≥2-fold in at least one of the macaque groups as compared to PBS-infected controls. The data presented are error-weighted averages. Genes shown in red were upregulated and in green downregulated in infected animals relative to PBS-infected animals (log (base 2) transformed expression values with minimim and maximum values of the color range being −4 and 4). Genes shown in grey were not significantly differentially regulated. See Table S2 and S4 for full gene names and expression values. (0.05 MB PDF) [file ppat.1000756.s008.pdf]

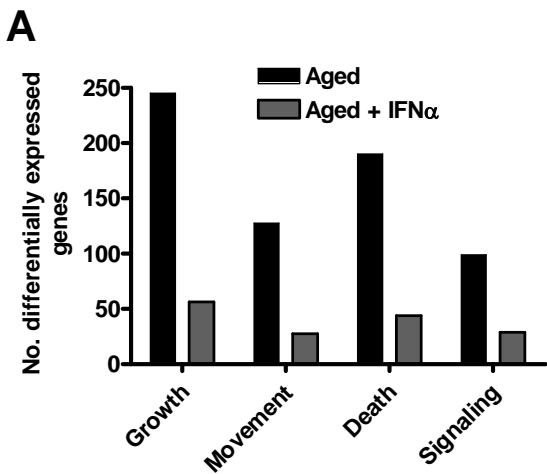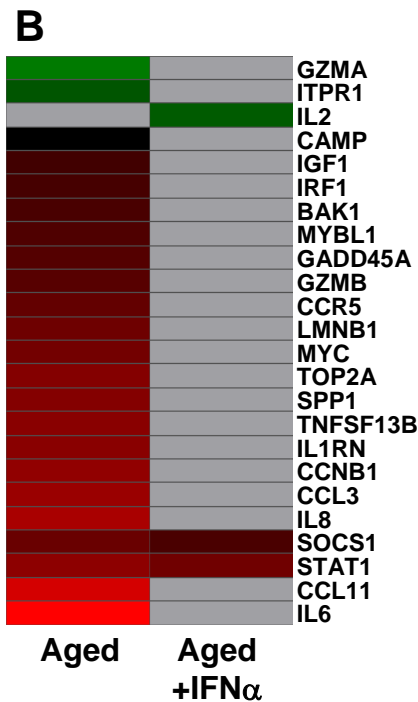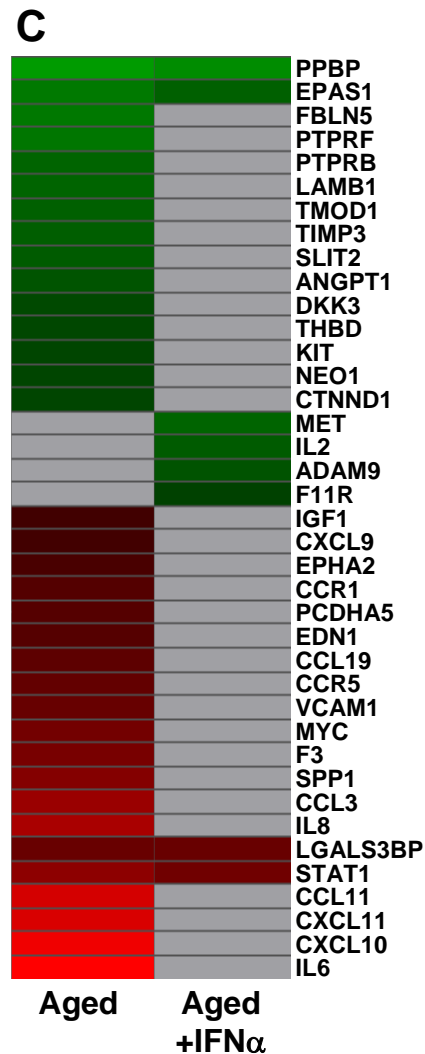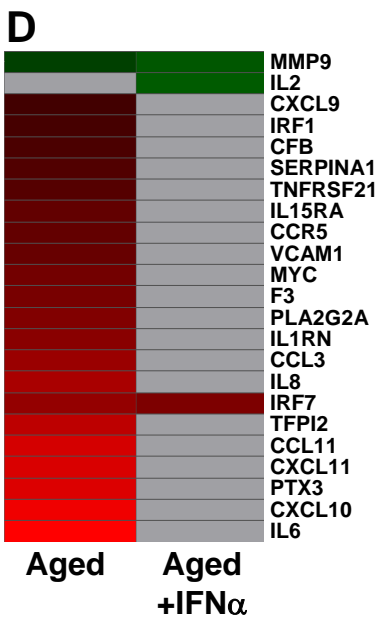

**Fig. S5**

Supplement: Figure S5 — Microarray analyses of the lower respiratory tract of SARS-CoV-infected aged and aged macaques treated with pegylated IFN-α. (A) Number of differentially expressed genes in macaque groups compared to PBS-infected animals with functions in cell growth and proliferation, cell movement, cell death, or cell-to-cell signaling and interaction obtained from Ingenuity Pathways Knowledge Base. When SARS-CoV-infected aged macaques were compared directly to IFN-treated aged macaques, these gene sets were significantly differentially expressed. (B–D) Gene expression profiles showing differentially expressed genes coding for proteins involved in apoptosis (B), cell adhesion (C), or NF-κB-signaling (D) of IFN-α-treated and untreated aged macaques. Genes displayed were obtained from Ingenuity Pathways Knowledge Base or literature and changed ≥2-fold in at least one of the groups as compared to PBS-infected controls. The data presented are error-weighted averages. Genes shown in red were upregulated, in green downregulated, and in grey not significantly differentially expressed in infected animals relative to PBS-infected animals (log (base 2) transformed expression values with minimum and maximum values of the color range being −4 and 4). Global test analysis of the direct contrast of SARS-CoV-infected aged versus IFN-treated aged animals showed that the cell adhesion and apoptosis pathways were significantly differentially expressed (p<0.05). See Table S2 and S3 for full gene names and expression values. (0.03 MB PDF) [file ppat.1000756.s009.pdf]
