# Supplementary material for: Exacerbated Innate Host Response to SARS-CoV in Aged Non-Human Primates
Source: PLoS Pathog. 2010 Feb 5;6(2):e1000756. doi: 10.1371/journal.ppat.1000756 (PMC2816697; doi:10.1371/journal.ppat.1000756)
Supplement: Figure S3 — Global gene expression profiles of individual young adult and aged animals. For a subset of gene transcripts, cytokines and chemokines, normalized log-2 based hybridization values for individual aged and young adult macaques are shown. (0.01 MB PDF) [file ppat.1000756.s007.pdf]

Gene symbol	Young adult						Aged					
	1	2	3	4	5	6	1	2	3	4	5	6
BMP1	6,62	6,93	6,88	6,68	7,22	7,06	6,15	6,23	6,24	6,63	6,62	7,14
CCL11	9,31	11,02	9,14	10,84	7,86	7,30	9,91	8,81	10,33	10,84	8,82	7,54
CCL19	7,41	6,97	7,97	8,50	7,14	6,15	7,36	8,06	7,58	7,99	8,28	8,29
CCL3	8,44	9,41	7,03	9,31	9,24	8,99	10,25	9,70	9,11	10,64	9,76	8,38
CCL4L1	8,05	8,85	6,87	8,75	8,82	8,80	9,52	8,53	8,61	9,49	8,90	8,13
CCL8	9,82	10,13	7,18	10,72	8,61	7,83	11,27	10,78	10,20	10,66	8,86	8,91
CXCL1 /// CXCL3	8,97	9,27	8,99	9,92	7,83	8,01	11,08	8,76	9,57	11,60	9,47	8,73
CXCL10	10,51	11,47	9,11	11,30	9,21	9,13	11,99	11,60	10,99	11,97	10,34	8,13
CXCL11	8,94	10,13	8,00	9,83	8,00	8,23	10,82	10,37	10,04	10,50	9,62	7,37
CXCL9	6,98	7,20	6,80	6,64	6,61	6,93	6,33	7,15	6,64	7,30	6,27	5,23
IFNB1	6,41	7,65	5,84	6,51	5,88	5,77	6,55	6,49	6,13	6,55	5,89	5,36
IL1RN	9,24	9,43	8,19	9,50	8,66	8,57	10,78	10,12	10,07	11,07	9,60	8,97
IL6	9,16	8,82	8,14	9,52	6,73	7,45	10,20	9,76	9,85	11,47	9,17	8,72
IL8	7,59	8,57	7,64	8,84	7,36	7,02	11,46	8,06	9,40	11,77	10,41	9,38
MCP-1	10,89	11,36	9,55	11,83	9,25	9,00	12,32	11,62	11,54	12,27	11,09	10,97
PPBP	5,53	5,34	5,43	5,35	5,88	6,58	5,31	7,02	5,13	5,44	5,25	5,58
SPP1	6,50	7,35	6,01	7,58	6,19	5,92	9,34	7,35	7,47	8,75	7,89	7,11
TNFSF13B	10,49	10,65	9,63	11,01	9,45	9,38	11,28	11,24	10,79	11,16	10,61	9,78

Fig. S3
